# CircSARS-CV2-N1368 from SARS-CoV-2 impairs endothelial cell function through the upregulation of ATF7 to activate TLR4/NF-κB/ROS signaling

**DOI:** 10.1038/s41401-025-01516-8

**Published:** 2025-03-11

**Authors:** Yi-hong Wen, Heng-li Zhao, Shao-yu Wu, Jia-xue Jiang, Yuan Gao, Zi-fan Wang, Xiao-yao Liu, Fei Yu, Tao Ou, An-zhi Zhao, Li-wen Chen, Jin-hua Fang, Hua-yan Wu, Jie-ning Zhu, Ning Ma, Jiu-feng Sun, Xian-hong Fang, Zhi-xin Shan

**Affiliations:** 1 School of Medicine, South China University of Technology, Guangzhou, 510006 China; 2 Medical Research Institute, Guangdong Provincial People’s Hospital, Guangdong Academy of Medical Sciences, Southern Medical University, Guangzhou, 510080 China; 3 School of Basic Medical Sciences, Guangzhou National Laboratory, Guangzhou Medical University, Guangzhou, 510005 China; 4 Guangdong Provincial Key Laboratory of Clinical Pharmacology, Guangdong Provincial People’s Hospital, Guangdong Academy of Medical Sciences, Southern Medical University, Guangzhou, 510080 China; 5 Guangdong provincial Institute of public health, Guangdong Provincial Center for Disease Control and Prevention, Guangzhou, 511430 China; 6 Guangdong Cardiovascular Institute, Guangdong Provincial People’s Hospital, Guangdong Academy of Medical Sciences, Southern Medical University, Guangzhou, 510080 China

**Keywords:** SARS-CoV-2, circular RNA, miR-103a-3p, endothelial cells, reactive oxygen species

## Abstract

SARS-CoV-2 can encode circular RNAs (circRNAs); however, the potential effects of exogenous SARS-CoV-2 circRNAs on cardiovascular sequelae remain unknown. Three circRNAs derived from the nucleocapsid (N) gene of SARS-CoV-2, namely, circSARS-CV2-Ns, were identified for functional studies. In particular, circSARS-CV2-N1368 was shown to enhance platelet adhesiveness to endothelial cells (ECs) and inhibit EC-dependent vascular relaxation. Moreover, exogenous expression of circSARS-CV2-N1368 suppressed EC proliferation and migration and decreased angiogenesis and cardiac organoid beating. Mechanistically, we elucidated that circSARS-CV2-N1368 sponged the microRNA miR-103a-3p, which could reverse circSARS-CV2-N1368-induced EC damage. Additionally, activating transcription factor 7 (ATF7) was identified as a target gene of miR-103a-3p, and Toll-like receptor 4 (TLR4) was verified as a downstream gene of ATF7 that mediates circARS-CV2-N1368-induced activation of nuclear factor kappa B (NF-κB) signaling and ROS production in ECs. Importantly, the reactive oxygen species (ROS) scavenger NAC mitigated the circSARS-CV2-N1368-promoted EC impairment. Our findings reveal that the TLR4/NF-κB/ROS signal pathway is critical for mediating circSARS-CV2-N1368-promoted oxidative damage in ECs, providing insights into the endothelial impairment caused by circSARS-CV2-Ns.

## Introduction

Severe acute respiratory syndrome coronavirus 2 (SARS-CoV-2), which is responsible for coronavirus disease 2019 (COVID-19), has spread across the world for nearly 4 years, resulting in various clinical presentations. In addition to the respiratory complications as a major feature, SARS-CoV-2 infection increases the risk of cardiovascular diseases (CVDs), such as myocarditis, acute coronary syndrome, right ventricular dysfunction, arrhythmias and thromboembolic complications [1, 2]. To date, the pathophysiological mechanisms of SARS-CoV-2-induced CVD are still poorly understood.

SARS-CoV-2 comprises a single-stranded positive-sense RNA encoding 4 structural proteins, including spike (S), envelope (E), membrane (M), and nucleocapsid (N) proteins, as well as sixteen nonstructural proteins and multiple accessory proteins [3]. Following the entry of SARS-CoV-2 into host cells, the positive-strand viral genomic (+) RNA is translated into viral polymerase proteins; subsequently, the negative-strand subgenomic (-) RNAs are synthesized and used as templates for subgenomic (+) messenger RNA (mRNA) synthesis [4]. During the replication of SARS-CoV-2 genomic RNA, several viral noncoding RNAs, including microRNAs (miRNAs), long noncoding RNAs (lncRNAs) and circular RNAs (circRNAs), can be generated coincidentally [5]. Approximately 90 miRNAs [6] and 354 [7], 351 [8], and 470 [9] circRNAs are predicted to be encoded by SARS-CoV-2. The generation of circRNAs encoded by SARS-CoV-2 has shown positive- and negative-strand bias, with the positive strand producing more circRNAs; moreover, circRNAs are highly expressed from certain regions of ORF1ab, M, ORF6/ORF7a/ORF7b and ORF8/N of SARS-CoV-2 [9].

CircRNAs are covalently closed at the 5’ and 3’ ends, resulting in high tissue-specific expression and RNA stability [10]. Notably, circRNAs perform their biological functions by acting as miRNA sponges, combining with RNA-binding proteins (RBPs), which have potential in regulating protein translation and gene transcription [11]. One study showed that the circRNA circ_3205 synthesized by SARS-CoV-2 sponges miR-298 to upregulate the expression of the KCNMB4 and PRKCE mRNAs, and these two genes are related to blood coagulation and the immune response [5]. Another report based on target gene prediction and a functional enrichment analysis suggested that SARS-CoV-2 circRNAs are closely related to cancer and have potential roles in regulating host cell functions [9]. Nevertheless, the expression patterns of SARS-CoV-2 circRNAs and their roles in SARS-CoV-2 infection and the diseases it causes remain unexplored.

In this study, we identified 5 circRNAs derived from the SARS-CoV-2 N gene, namely, circSARS-CV2-Ns. Three SARS-CV2-Ns, circSARS-CV2-N654, -N804, and -N1368, were exogenously overexpressed in human cardiac microvascular endothelial cells (HCMECs). Functionally, the overexpression of these 3 circRNAs markedly aggravated platelet adhesiveness to HCMECs, increased reactive oxygen species (ROS) levels, inhibited HCMEC proliferation and migration, and attenuated angiogenesis in vitro and in vivo. Notably, circSARS-CV2-N1368 more significantly inhibited EC-dependent vascular relaxation and CO beating. Moreover, we determined that circSARS-CV2-N1368 could specifically sponge miR-103a-3p to activate the activating transcription factor 7 (ATF7)–Toll-like receptor 4 (TLR4)–nuclear factor kappa B (NF-κB) signaling pathway to increase ROS production. The ROS scavenger NAC abolished the impairment of HCMECs overexpressing circSARS-CV2-N1368. In essence, TLR4/NF-κB/ROS, as critical signals, mediate circSARS-CV2-N1368-promoted oxidative damage to vascular endothelial cells.

## Materials and methods

### Ethics statement

The pharyngeal swab samples from patients with COVID-19 were provided by the Guangdong Provincial Center for Disease Control and Prevention. Male C57BL/6 mice (24 ± 3 g) and Sprague‒Dawley (SD) rats (200–250 g) were purchased from the Department of Experimental Animal Research Center, Sun Yat-sen University, Guangzhou, China (license no. SCXK [YUE] 2004-0011). The mice were housed on a 12-h light/dark cycle under pathogen-free conditions with free access to standard mouse chow and tap water. This study conformed to the Guide for the Care and Use of Laboratory Animals published by the US National Institutes of Health (8th Edition, National Research Council, 2011). The present study was also approved by the Research Ethics Committee of Guangdong Provincial People’s Hospital.

### Preparation of blood serum from hACE2 transgenic mice with SARS-CoV-2 infection

The methodology was performed according to the protocol in a previous report [12].

### Antibodies, reagents and resources

The detailed information is provided in Supplemental Table S1.

### Cell culture and cell lines

Neonatal mouse ventricular cardiomyocytes (NMVCs) and cardiac fibroblasts (CFs) were isolated from the hearts of 1- to 3-day-old newborn C57BL/6 mice and cultured as previously described [13, 14].

Human cardiac microvascular endothelial cells (HCMECs) were cultured in an iCell primary endothelial cell culture system (iCell Bioscience Inc., Shanghai, China), while human embryonic kidney 293 (HEK293) and cardiomyocyte AC16 cells were cultured in DMEM with 10% fetal bovine serum (Zeta LIFE, USA) at 37 °C with 5% CO_2_.

### RNA isolation and quantitative real-time polymerase chain reaction (RT‒qPCR)

Total RNA was extracted from pharyngeal swab samples from patients with COVID-19, HCMECs, COs and mouse blood serum with TRIzol reagent (Invitrogen), followed by cDNA synthesis and real-time quantitative PCR (RT‒qPCR) assays, as described in our previous report [15]. The 2^-ΔCT^ and 2^-ΔΔCT^ methods were used to calculate the expression levels of the relevant coding genes, circSARS-CV2-Ns and the corresponding miRNAs. The PCR primers used in this study and the sizes of the amplified DNA fragments are shown in Supplemental Table S2.

### Western blot analysis

Cells and SD rat coronary arteries were lysed with RIPA lysis buffer containing protease inhibitors (Roche; 04693132001) for protein extraction, followed by Western blot analysis [14]. The membranes containing the target proteins were labeled with primary antibodies against ANP, β-MHC, α-SMA, p-e-NOS, e-NOS, VEGFA (Abcam), COL1A1, COL3A1, ATF7, TLR4, p-NF-κB p65, NF-κB p65, GAPDH (Proteintech), p-HH3, and HH3 (Cell Signaling Technology). Horseradish peroxidase-conjugated secondary antibodies and the chemiluminescence system were obtained from Millipore (Millipore, MA, USA). Band intensities were quantified using the digital densitometry tool of ImageJ software, and the intensity of the GAPDH band was used as a loading control.

### Cell proliferation and migration

According to the manufacturer’s instructions, EdU staining (RiboBio, Guangzhou, China) was conducted to determine the proliferative capacity of the HCMECs. A transwell migration assay was performed to assess the migration capacity of the HCMECs. Briefly, HCMECs were cultured in the upper chamber with a relatively high concentration of fetal bovine serum in the medium in the lower chamber. The number of cells that migrated to the lower chamber was counted to evaluate the migratory capacity of the HCMECs.

A wound healing assay was also performed to determine the migratory capacity of the HCMECs. HCMECs were cultured and infected with the recombinant circSARS-CV2-N adenovirus, and the cells on the plate were then scratched with a sterile pipette tip, followed by another 24-h culture. Images were collected at 0 h and 24 h after adenovirus infection using an inverted light microscope.

### Construction of adenovirus vectors for the exogenous expression of circSARS-CV2-Ns

The DNA fragments corresponding to circSARS-CV2-N654, -N804, and -N1368 and their flanking sequence of approximately 200 nt in length were synthesized and inserted into the multiple cloning site in the pAd-Track-cmv vector (Coloncancer, USA). The recombinant pAd-Track-cmv-circRNAs were subsequently used to construct the recombinant adenovirus plasmid with the pAdEasy-I (Coloncancer, USA) plasmid in BJ5183 *Escherichia coli*. Finally, the recombinant circRNA adenovirus plasmid was linearized with the restriction enzyme *Pac* I and transfected into 293 T cells to package the recombinant circRNA adenovirus. Moreover, a control adenovirus expressing green fluorescent protein (GFP), rAd-GFP, was also prepared.

### Dual luciferase assay

A recombinant pGL3-promoter luciferase reporter plasmid containing the potential miR-103a-3p binding site sequence in circSARS-CV2-N1368 was constructed to verify the interaction between miR-103a-3p and circSARS-CV2-N1368. A recombinant pGL3-enhancer luciferase reporter plasmid, which contains the DNA sequence of the human TLR4 gene promoter region and is 1.5 kb in length, was also constructed to investigate the ability of ATF7 to bind the TLR4 gene promoter. The luciferase reporter assay was performed using HEK293 cells, and the luciferase activities were determined using the dual luciferase reporter assay system (Promega, USA) according to the manufacturer’s protocol.

### RNA Pull‑Down Assay

Adenovirus-mediated overexpression of circSARS-CV2-N1368 was achieved in HCMECs. The biotin-labeled circSARS-CV2-N1368 probe (RiboBio, China) was transfected into human cardiomyocyte AC16 cells. The cell lysates were incubated with streptavidin beads (BersinBio, Guangzhou, China) at 25 °C for 40 min. After washes with wash buffer, the RNA complexes bound to the beads were eluted, followed by extraction with an RNeasy Mini Kit (QIAGEN, Germany) and detection of the potentially sponged miRNAs.

### Human cardiac organoid differentiation

For the differentiation of human iPSCs into cardiac organoids (COs), a previously published protocol was adopted, with some modifications [16]. Briefly, hPSCs were grown to 60%–90% confluence and dissociated using Accutase (Sigma, USA) to obtain a single-cell mixture. A total of 10,000 cells were seeded into each well of a round bottom ultralow attachment 96-well plate in a volume of 100 μL. The plate was centrifuged at 200 × *g* for 5 min. After 24 h, 50 μL of medium was removed, and fresh Essential 8 Medium (Gibco, USA) was added. After 48 h (Day 0), the medium was changed to RPMI-1640/B27 medium without insulin (Gibco) containing CHIR99021, BMP4, and activin A. On Day 2, the medium was replaced with RPMI/B27 minus insulin with IWR1 (Selleck). On Day 6, the medium was changed to RPMI/B27 (with insulin). On Day 7, a second CHIR99021 induction was conducted for 1 h. The medium was replaced every 48 h thereafter with RPMI/B27 medium. After 30 days, COs were collected for further experimental studies.

### Matrigel-based tube formation in vitro and in vivo

Tube formation in vitro was assessed using a Matrigel kit (BD Biosciences, CA, USA) according to the manufacturer’s instructions. Briefly, 250 μl of thawed ECM gel solution mixed with 250 µl of DMEM/F12 was added to each well of a 12-well plate and incubated for 1 h at 37 °C. The wells were subsequently seeded with 1 mL of an endothelial cell suspension containing 5×10^6^ cells. The cells were cultured at 37 °C for 6 h, and the development of tubular structures was examined in situ using an inverted light microscope.

For the in vivo Matrigel plug assay, 5×10^5^ endothelial cells were mixed with 0.4 mL of cold Matrigel and injected into the subcutaneous tissues of the mice. After injection, the Matrigel rapidly formed a solid plug at the site of injection. Seven days later, the Matrigel plugs were excised, and the microvessel density was evaluated via a microscopic analysis after hematoxylin and eosin (HE) staining.

### Platelet adhesion assay

HCMECs were seeded on flow chamber slides (u-slides, Ibidi-Integrated BioDiagnostics, Martinsried, Germany) and grown until they reached confluence. Then, the cells were infected with adenoviruses carrying circSARS-CV2-N654, -N804, -N1368, and the efficiency of adenovirus infection in HCMECs was evaluated by detecting the coexpression of green fluorescent protein (GFP). Moreover, human platelets were separated from citrate anticoagulant-treated whole blood. Blood was centrifuged at 200 × *g* for 10 min at room temperature to obtain platelet-rich plasma (PRP). The PRP was then perfused with a shear rate of 500 s^-1^. Images were collected at 0, 10, and 20 min postperfusion using a Nikon Ti2-U inverted microscope, and ImageJ software was used to count the number of aggregated platelets.

### Vessel preparation and force measurement

SD rats were anesthetized with isoflurane and subsequently euthanized with cervical dislocation. The hearts were excised and transferred to a dissection dish filled with 4 °C Krebs–Henseleit (K-H) solution. The coronary arteries were dissected from the connective tissues under a stereomicroscope in an ice bath. The vessels were cut into ring segments of approximately 2 mm in length, followed by an overnight infection with circSARS-CV2-N654, -N804, -N1368.

The vascular rings were fixed in a multi myograph system (Danish Myo Technology, Aarhus, Denmark), and the vascular tone was recorded. Approximately 5 mL of K-H solution was added, and the vascular rings were oxygenated continuously with a gas mixture (95% O_2_ and 5% CO_2_). After the vascular rings were balanced for 30 min, the blood vessels were stretched open with a basal tension of 1.5 mN and balanced for 60 min. During this period, the rings were exposed to a K-H solution containing high potassium (60 mM K^+^) every 15 min to detect the reactivity of the arterial tone. When the arterial tone achieved the maximal contractile response, the vessels were washed with K-H solution 4 times to restore the baseline tone.

Vascular reactive equilibrium was reached when the difference in the arterial contractile response caused by the high-potassium solution between two consecutive reactions was less than 10%. After the addition of 1 μM 5-HT to shrink the vessel rings to the maximum value, a cumulative concentration administration assay was performed. The vascular endothelial relaxant acetylcholine (1 nM, 3 nM, 10 nM, 30 nM, 100 nM, 300 nM, 1 µM, 3 µM, and 10 µM) was added to the bath solution to determine the endothelial function of the vascular rings.

### Statistical analysis

The results are presented as the means ± standard deviation (SD) of at least three independent experiments and were analyzed using GraphPad Prism 9 (La Tolla, CA, USA). For the analysis of differences between two groups, Student’s *t* test was performed. One-way or 2-way ANOVA followed by a *post hoc* test (Bonferroni) were chosen for the comparisons of interest without adjustment for multiple comparisons. A *P* value < 0.05 was considered statistically significant.

## Results

### Validation of circRNAs derived from the SARS-CoV-2 N gene

**Fig. 1:**
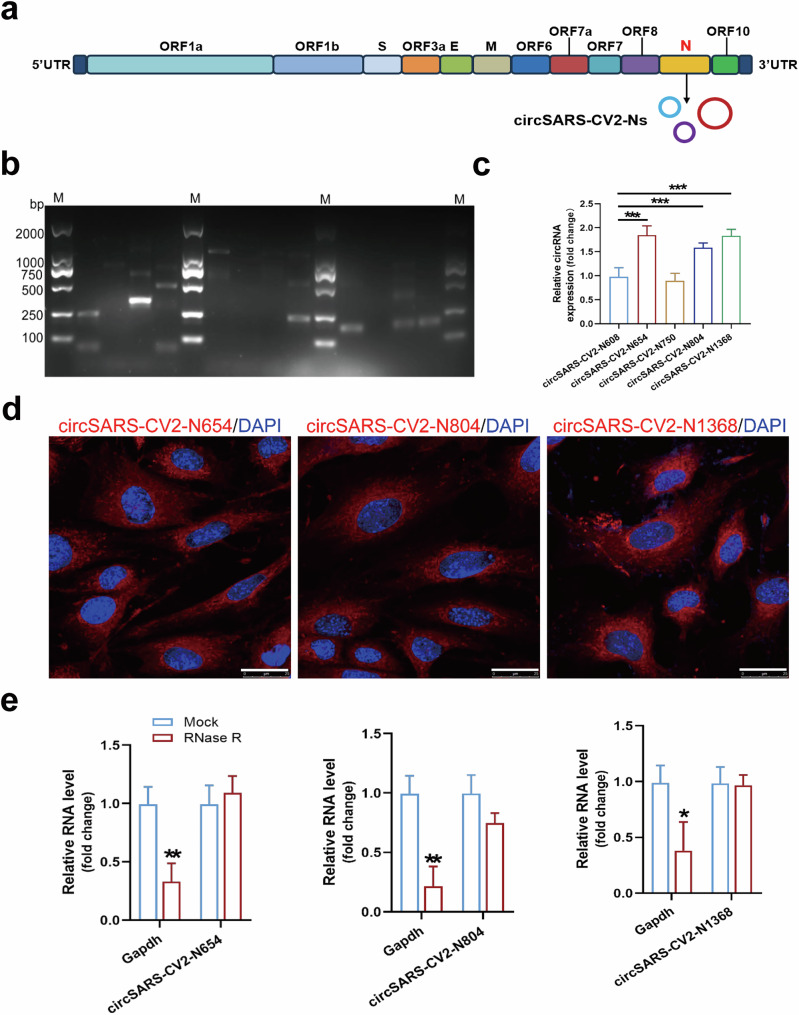
Identification of circRNAs derived from SARS-CoV-2 nucleocapsid gene. **a** Genome organization of SARS-CoV-2, and circSARS-CV2-Ns generated from SARS-CoV-2 N gene. **b** PCR products of circSARS-CV2-Ns by agarose gel electrophoresis. **c** Detection of SARS-CV2-Ns in pharyngeal swab samples from patients with COVID-19 by RT-qPCR assay (1-way ANOVA, *n* = 5 per group). **d** RNA FISH assay of circSARS-CV2-N654, -N804, and -N1368 in human cardiomyocyte AC16. CircRNAs were shown in red and nuclei were stained with DAPI. The scale bar is 25 μm. **e** Levels of circSARS-CV2-N654, -N804, -N1368 and GAPDH mRNA in RNase R-treated total RNA (20 U/mg RNA) from AC16 cells were detected by RT-qPCR assay (unpaired *t* test, *n* = 3 per group). *P < 0.05; **P < 0.01; ***P < 0.001.

In the present study, circSARS-CV2-Ns derived from the SARS-CoV-2 N gene were identified and their functions were explored (Fig. 1a). Using sets of divergent PCR primers, the PCR products of potential circSARS-CV2-Ns were amplified from pharyngeal swab samples from patients with COVID-19, and the junction site sequences of potential circRNAs were identified by a Sanger DNA sequencing assay. Nucleotide sequences of 5 circSARS-CV2-Ns were predicted; in particular, circSARS-CV2-N1368 also covers the sequence of the SARS-CoV-2 ORF8 gene (Fig. 1b, Supplemental Fig. S1). RT‒qPCR revealed that the levels of circSARS-CV2-N654, -N804, and -N1368 were relatively high among the above 5 circSARS-CV2-Ns detected in pharyngeal swab samples from patients with COVID-19 (Fig. 1c); thus, these 3 circRNAs were selected for further functional studies. Moreover, circSARS-CV2-N654 and -N1368, but not circSARS-CV2-N804, were detectable in the peripheral blood serum of humanized ACE2 (hACE2) transgenic mice infected with SARS-CoV-2 (Supplemental Fig. S2).

The results of the fluorescence in situ hybridization (FISH) assay revealed the predominant cytoplasmic distribution of circSARS-CV2-N654, -N804, -N1368 in human cardiomyocyte AC16 cells with exogenous overexpression of these circRNAs (Fig. 1d). We detected the stability of circSARS-CV2-N654, -N804, and -N1368 in AC16 cells. The RT‒qPCR results indicated that these circRNAs were efficiently resistant to RNase R treatment (Fig. 1e). Overall, we identified the existence and characteristics of circRNAs derived from the SARS-CoV-2 N gene in patients with COVID-19.

### Overexpression of circSARS-CV2-Ns enhances platelet adhesiveness

**Fig. 2:**
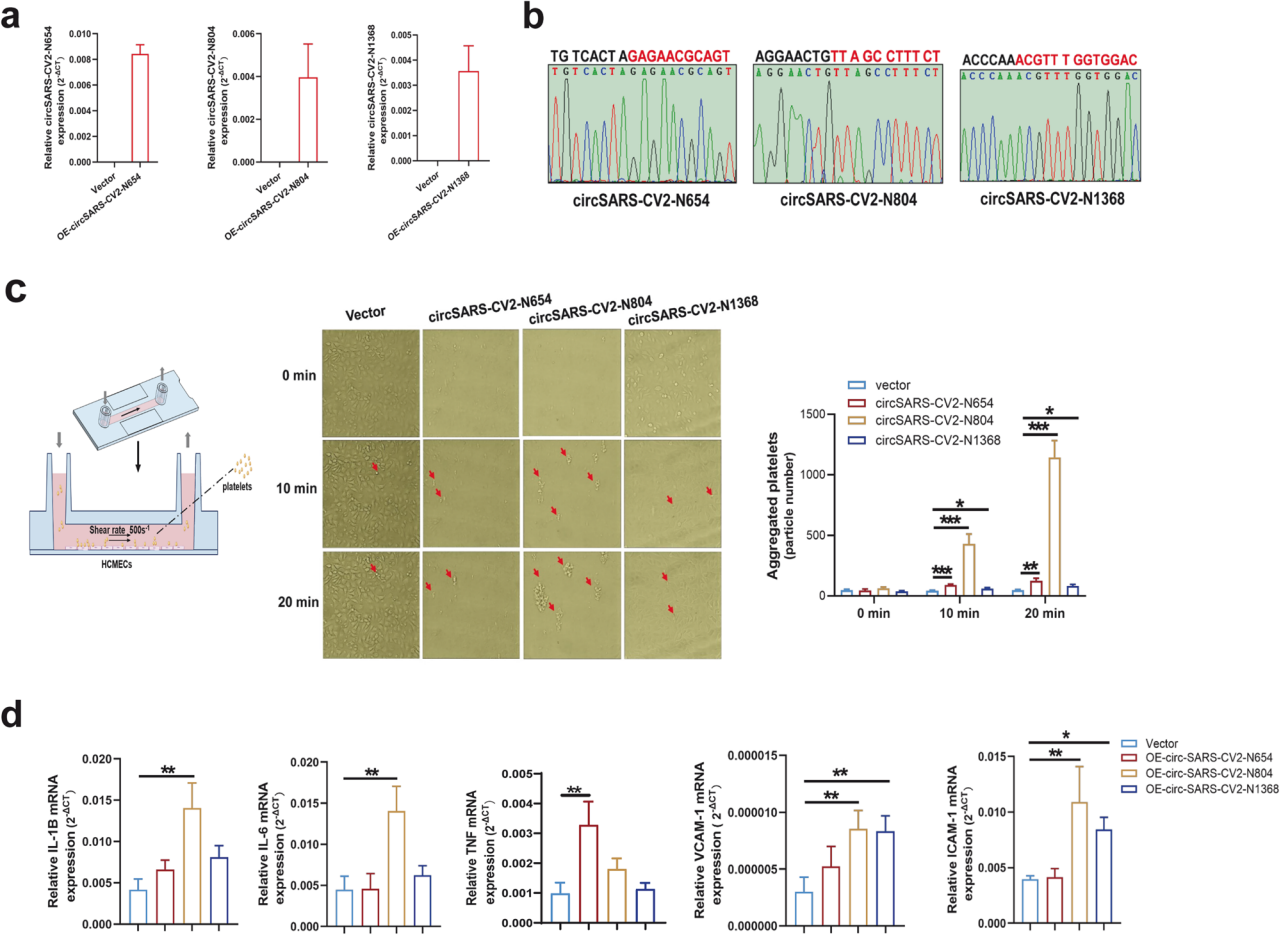
Overexpression of circSARS-CV2-Ns enhances platelet adhesiveness onto HCMECs. **a** Detection of SARS-CV2-Ns overexpression in HCMECs by RT-qPCR assay. **b** Identification of the junction site sequence of adenovirus-mediated expression of circSARS-CV2-N654, -N804, and -N1368 by Sanger DNA sequencing assay. **c** Overexpression of circSARS-CV2-N654, -N804, -N1368 promoted platelet adhesiveness onto HCMECs (1-way ANOVA, *n* = 3 per group). **d** MRNA expression of inflammatory-related genes in HCMECs by RT-qPCR assay (1-way ANOVA, *n* = 3 per group). *P < 0.05; **P < 0.01; ***P < 0.001.

The effects of circSARS-CV2-Ns on platelet adhesiveness to endothelial cells were investigated by exogenously overexpressing circSARS-CV2-Ns in HCMECs. The results of RT‒qPCR and Sanger sequencing revealed that the adenovirus-mediated overexpression of circSARS-CV2-N654, -N804, and -N1368 was successfully achieved in HCMECs (Fig. 2a, b).

The results of the platelet adhesion assay showed that circSARS-CV2-N654, -N804, and -N1368 could enhance platelet adhesion to HCMECs; in particular, circSARS-CV2-N804 could markedly promote platelet adhesion to HCMECs (Fig. 2c). The mRNA expression of inflammation-related genes, including IL-1B, IL-6, VCAM-1 and ICAM-1, was markedly upregulated in circSARS-CV2-N804-overexpressing HCMECs, and the mRNA expression of VCAM-1 and ICAM-1 was also significantly increased in circSARS-CV2-N1368-overexpressing HCMECs (Fig. 2d). Together, these results indicate that exogenous expression of circSARS-CV2-N654, -N804, -N1368 can enhance platelet adhesiveness to HCMECs and increase the expression of inflammation-related genes in HCMECs.

### CircSARS-CV2-Ns impair endothelium-dependent vasorelaxation and endothelial cell function

**Fig. 3:**
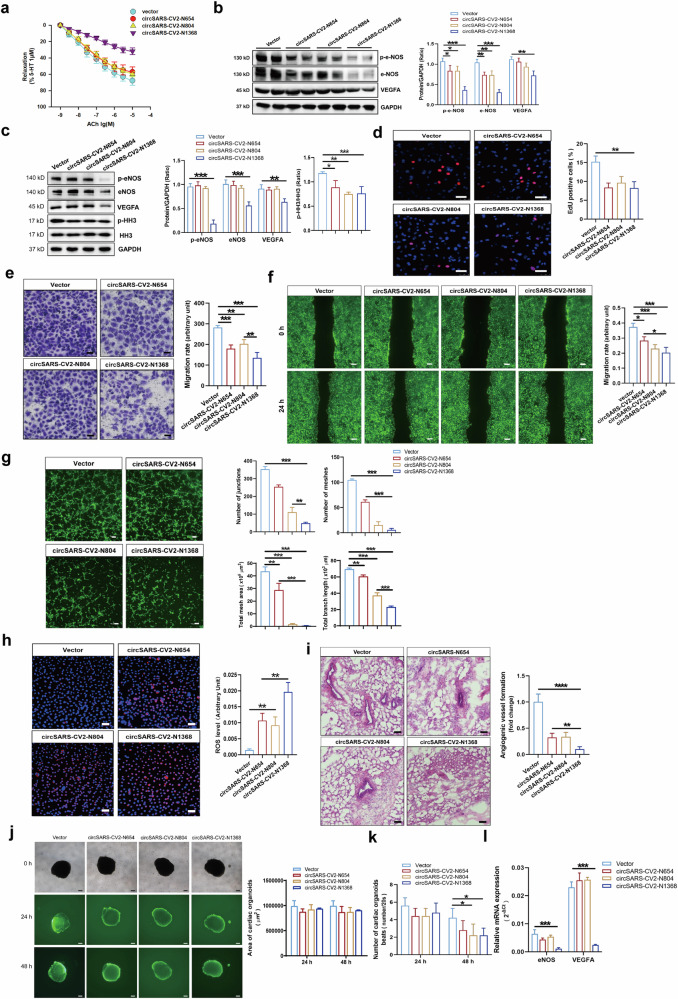
Effects of circSARS-CV2-Ns overexpression on endothelium-dependent vasorelaxation and phenotypes of HCMECs. **a** Detection of acetylcholine-induced endothelium-dependent vascular relaxation in rat coronary arteries with overexpression of circSARS-CV2-Ns (1-way ANOVA, *n* = 4 per group). **b** VEGFA expression and e-NOS activation (eNOS(S1177) phosphorylation) in rat coronary arteries by Western blot assay. **c** VEGFA expression, e-NOS and HH3 activation (phosphorylation of HH3) in circSARS-CV2-Ns-overexpressiong HCMECs by Western blot assay (1-way ANOVA, *n* = 3 per group). **d** Proliferation activity analysis of HCMECs by EdU assay (1-way ANOVA, *n* = 3 per group). Migration activity analysis of HCMECs by trans-well migration assay (**e**) and wound healing assay (**f**) (1-way ANOVA, *n* = 3 per group), respectively. **g** Microscopic images showing matrigel tube formation of HCMECs with overexpression of circSARS-CV2-Ns (1-way ANOVA, *n* = 3 per group). **h** Detection of ROS level by using DCFH-DA probe (1-way ANOVA, *n* = 3 per group). **i** Effect of exogenous overexpression of circSARS-CV2-Ns on angiogenesis in vivo by matrigel plug assay (1-way ANOVA, *n* = 5 per group). **j**, **k** Effects of exogenous overexpression of circSARS-CV2-Ns on size and beat of COs (2-way ANOVA, *n* = 5 per group). **l** Detection of eNOS and VEGFA mRNA expression by RT-qPCR assay (2-way ANOVA, *n* = 3 per group). The scale bar is 50 μm in (**d**), (**e**), (**h**), (**i**), the scale bar is 100 μm in (**g**), the scale bar is 200 μm in (**f**), (**j**). *P < 0.05; **P < 0.01; ***P < 0.001; ****P < 0.0001

We determined the endothelial function of SD rat coronary arteries with exogenous overexpression of circSARS-CV2-Ns. The results of the force measurement assay revealed that circSARS-CV2-N1368 overexpression markedly inhibited the endothelial cell-dependent diastolic response and decreased eNOS activation and VEGFA expression in rat coronary arteries (Fig. 3a, b, Supplemental Fig. S3). Moreover, Western blot results showed that the overexpression of circSARS-CV2-N654, -N804, and -N1368 markedly suppressed histone H3 (HH3) activation, induced more significant decreases in VEGFA expression and e-NOS activation, as well as decreased the levels of the eNOS dimer in circSARS-CV2-N1368-overexpressing HCMECs (Fig. 3c, Supplemental Fig. S4).

Next, we investigated the effects of circSARS-CV2-N654, -N804, -N1368 on the proliferation and migration of HCMECs. EdU assays revealed that the exogenous expression of circSARS-CV2-N654, -N804, and -N1368 inhibited the proliferation of HCMECs (Fig. 3d). Consistently, the results of the transwell migration and wound healing assays revealed that the overexpression of circSARS-CV2-N654, -N804, and -N1368 suppressed HCMEC migration and that circSARS-CV2-N1368 strongly inhibited HCMEC migration (Fig. 3e, f). Moreover, we found that the overexpression of circSARS-CV2-N654, -N804, and especially circSARS-CV2-N 1368 inhibited the ability of HCMECs to form tube structures (Fig. 3g).

We detected the reactive oxygen species (ROS) level in HCMECs and found that exogenous expression of circSARS-CV2-N654, -N804, and especially circSARS-CV2-N1368 markedly increased the total ROS level, in parallel with mitochondrial ROS, in HCMECs (Fig. 3h, Supplemental Fig. S5). Additionally, the results of the Matrigel plug assay revealed that the blood capillary density was decreased in Matrigel plugs mixed with HCMECs overexpressing circSARS-CV2-N654, -N804, or -N1368. In particular, in vivo angiogenesis was markedly attenuated in Matrigel plugs mixed with circSARS-CV2-N1368-overexpressing HCMECs (Fig. 3i).

Adenovirus-mediated overexpression of circSARS-CV2-N654, -N804, and -N1368 was achieved in neonatal mouse ventricular cardiomyocytes (NMVCs) and neonatal mouse cardiac fibroblasts (mCFs) to explore the effects of circSARS-CV2-N654, -N804, and -N1368 on the phenotypes of cardiomyocytes and cardiac fibroblasts. No significant changes in the expression of hypertrophy-related genes, including β-MHC and ANP, or HH3 activation were observed in NMVCs overexpressing circSARS-CV2-N654, -N804, or -N1368 (Supplemental Fig. S6). Similarly, no significant changes in the expression of fibrosis-related genes, including COL1A1, COL3A1 and α-SMA, or HH3 activation were observed in mCFs with exogenous overexpression of circSARS-CV2-N654, -N804, or -N1368 (Supplemental Fig. S7).

In the present study, adenovirus-mediated overexpression of circSARS-CV2-N654, -N804, and -N1368 was also achieved in human COs (COs). No significant changes in the size of COs were observed at 48 h after circSARS-CV2-N654, -N804, or -N1368 overexpression in COs (Fig. 3j). However, the number of beats was markedly reduced in COs overexpressing circSARS-CV2-N804 or -N1368 (Fig. 3k). RT‒qPCR revealed that the mRNA expression of eNOS and VEGFA was significantly decreased in circSARS-CV2-N1368-overexpressing COs (Fig. 3l). Moreover, the mRNA expression of NPPA and COL3A1 was increased in COs overexpressing circSARS-CV2-N654 and -N804 (Supplemental Fig. S8). Together, these results indicate that circSARS-CV2-Ns, especially circSARS-CV2-N1368, can increase ROS generation in ECs and impair EC function.

### CircSARS-CV2-N1368 specifically sponges miR-103a-3p in HCMECs

**Fig. 4:**
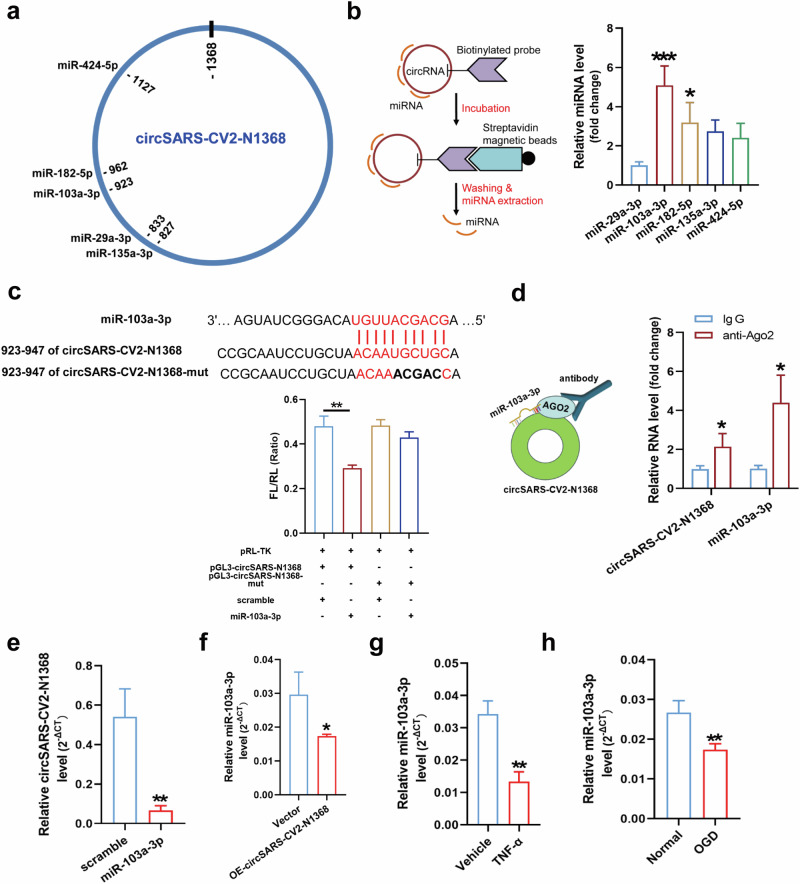
Validation of the interaction between circSARS-CV2-N1368 and miR-103a-3p. **a** The potential binding sites of 5 miRNAs in circSARS-CV2-N1368. **b** Identifcation of the interactions between 5 miRNAs and circSARS-CV2-N1368 by RNA pull-down assay (1-way ANOVA, *n* = 3 per group). **c** Identification of the binding site of miR-103a-3p in circSARS-CV2-N1368 by dual luciferase assay (1-way ANOVA, *n* = 3 per group). **d** RIP assay was performed by using anti-AGO2 antibody, and the enrichment of circSARS-CV2-N1368 and miR-103a-3p was determined by RT-qPCR assay. **e** Detection of circSARS-CV2-N1368 level in HCMECs with transfection of miR-103a-3p. **f** Detection of miR-103a-3p level in HCMECs with overexpression of circSARS-CV2-N1368, and in HCMECs exposed to TNF-α (**g**) and OGD treatment(**h**), respectively. Comparisons were made with unpaired *t* test, *n* = 3 per group in (**d)**–(**h**). *P < 0.05, **P < 0.01, ***P < 0.001.

Since circSARS-CV2-N1368 is located mainly in the cytoplasm, we explored potential miRNAs that can bind to circSARS-CV2-N1368 in HCMECs. Five miRNAs, including miR-29a-3p, -103a-3p, -135a-3p, -182-5p, and -424-5p, were predicted to bind circSARS-CV2-N1368 by two software programs, miRanda (www.microrna.org/microrna/home.do) and TargetScan (www.targetscan.org/) (Fig. 4a). An RNA pull-down assay revealed that miR-103a-3p and miR-182-5p could be efficiently sponged by circSARS-CV2-N1368 (Fig. 4b). The results of the dual-luciferase assay revealed that miR-103a-3p could specifically bind to circSARS-CV2-N1368 (Fig. 4c). Moreover, miR-103a-3p was verified as a highly expressed miRNA in the human brain, artery, veins and heart (Supplemental Fig. S9).

Since Argonaute 2 (AGO2) participates in small RNA-guided gene silencing processes [17], we conducted an RNA binding protein immunoprecipitation (RIP) assay and found that miR-103a-3p and circSARS-CV2-N1368 could be jointly pulled down by AGO2 in circSARS-CV2-N1368-overexpressing HCMECs, indicating that the binding of circSARS-CV2-N1368 to miR-103a-3p was mediated by AGO2 (Fig. 4d). Moreover, the transfection of miR-103a-3p decreased the level of exogenously expressed circSARS-CV2-N1368 in HCMECs, and the level of miR-103a-3p was also reduced in circSARS-CV2-N1368-overexpressing HCMECs (Fig. 4e, f). Additionally, a decrease in miR-103a-3p expression occurred in HCMECs subjected to oxygen‒glucose deprivation (OGD) or TNF-α protein treatment (Fig. 4g, h), indicating that miR-103a-3p may participate in EC impairment. Therefore, these results reveal that circSARS-CV2-N1368 specifically sponges and decreases the level of miR-103a-3p in ECs.

### CircSARS-CV2-N1368 impairs EC function by sponging miR-103a-3p

**Fig. 5:**
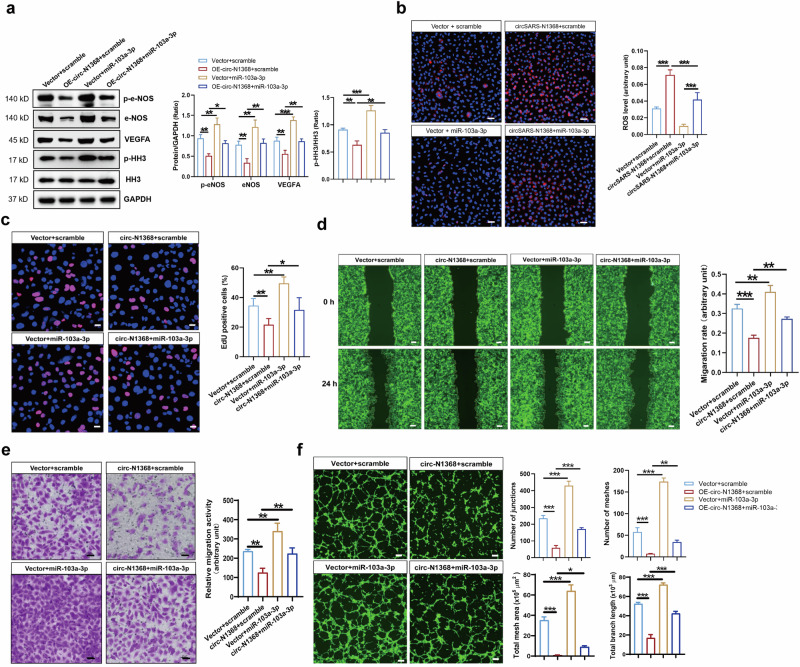
CircSARS-CV2-N1368 inhibits proliferation, migration and tube-formation of HCMECs via sponging miR-103a-3p. **a** Effect of miR-103a-3p on expression of VEGFA, e-NOS and HH3 activation in circSARS-CV2-N1368-overexpressiong HCMECs by Western blot assay. **b** MiR-103a-3p decreased ROS level in circSARS-CV2-N1368 overexpressing HCMECs. **c** Proliferation activity analysis of HCMECs by EdU assay. **d**, **e** Migration activity analysis of HCMECs by trans-well migration assay and wound healing assay, respectively. **f** Matrigel tube formation assay of circSARS-CV2-Ns-overexpressiong HCMECs with transfection of miR-103a-3p. Comparisons were made with 1-way ANOVA, *n* = 3 per group in (**b**)–(**f**). The scale bar is 50 μm in (**b**), **(c**), (**e**), the scale bar is 100 μm in (**f**), the scale bar is 200 μm in (**d**). *P < 0.05, **P < 0.01, ***P < 0.001.

We investigated the effect of miR-103a-3p on the circSARS-CV2-N1368-induced EC impairment. The Western blot results showed that miR-103a-3p rescued the decrease in VEGFA levels and inactivation of e-NOS and HH3 in circSARS-CV2-N1368-overexpressing HCMECs (Fig. 5a). The ROS level was increased in circSARS-CV2-N1368-overexpressing HCMECs, but miR-103a-3p reversed the increase in the ROS level (Fig. 5b). EdU assays revealed that miR-103a-3p reversed the inhibitory effect of circSARS-CV2-N1368 on HCMEC proliferation (Fig. 5c). Consistently, transwell migration and wound healing assays revealed that miR-103a-3p reversed the decrease in the migratory capacity of HCMECs caused by circSARS-CV2-N1368 (Fig. 5d, e). Additionally, miR-103a-3p abolished the inhibitory effect of circSARS-CV2-N1368 on the tube formation of HCMECs (Fig. 5f). Together, these data suggest that the sponging of miR-103a-3p mediates the ability of circSARS-CV2-N1368 to impair EC function.

### CircSARS-CV2-N1368 impairs EC dysfunction by targeting the miR-103a-3p/ATF7 axis

**Fig. 6:**
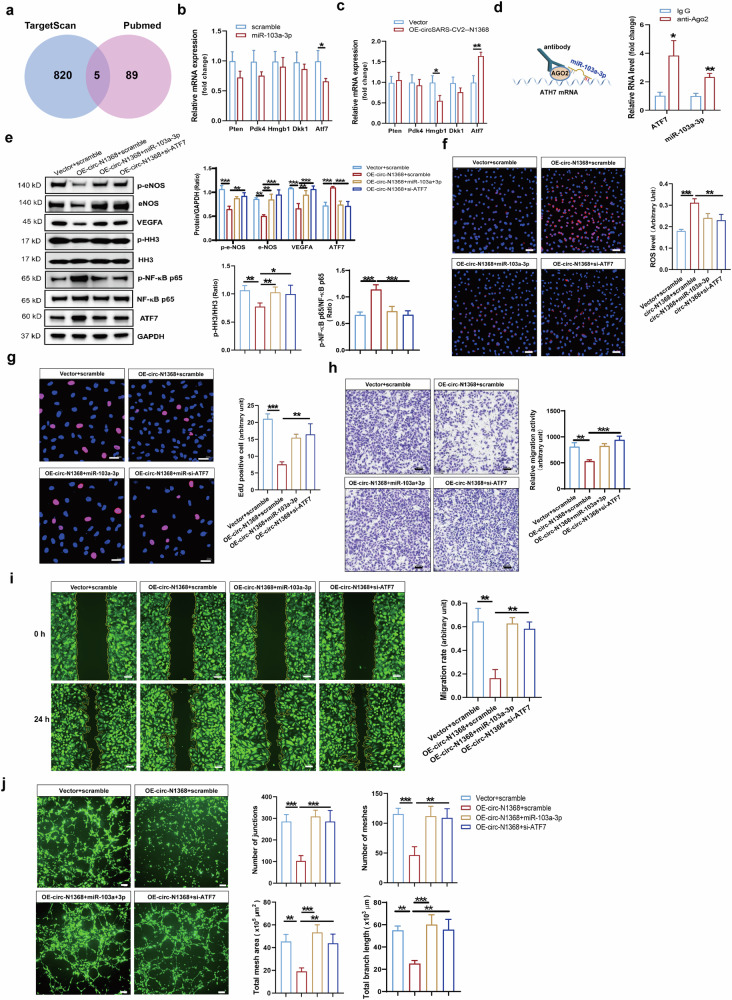
CircSARS-CV2-N1368 regulates the phenotypes of HCMECs by targeting miR-103a-3p/Atf7 axis. **a** Venn diagram showing the overlapped genes. **b** and **c** The expression of the concerned genes in HCMECs with transfection of miR-103a-3p by RT-qPCR assay. **d** Validation of the interaction between miR-103a-3p and Atf7 mRNA by RIP assay. **e** Determination of ATF7 and VEGFA expression, activations of e-NOS, HH3 and NF-κB p65 in HCMECs by Western blot assay. **f** MiR-103a-3p and si-Atf7 decreased ROS generation in circSARS-CV2-N1368 overexpressing HCMECs. **g** Proliferation activity analysis of HCMECs by EdU assay. **h, i** Migration activity of HCMECs by trans-well migration assay and wound healing assay, respectively. **j** Matrigel tube formation assay of circSARS-CV2-Ns-overexpressiong HCMECs with transfection of miR-103a-3p or si-Atf7. Comparisons were made with unpaired *t* test in (**b**)–(**d**), and comparisons were made with 1-way ANOVA in (**e**)–(**i**), *n* = 3 per group. The scale bar is 50 μm in (**f**)–(**h**), the scale bar is 100 μm in **i**, the scale bar is 200 μm in (**j**). **P < 0.01, ***P < 0.001.

Intersectional analyses of the total potential target genes based on TargetScan program and the target genes of miR-103a-3p reported in the PubMed database were performed, and 5 candidate genes, Pten, Pdk4, Hmgb1, Dkk1 and Atf7, were screened (Fig. 6a). The results of the RT‒qPCR assay revealed that Atf7 expression was significantly decreased in HCMECs transfected with miR-103a-3p but was markedly increased in circSARS-CV2-N1368-overexpressing HCMECs (Fig. 6b, c). RIP assays revealed that miR-103a-3p and the Atf7 mRNA were efficiently pulled down by the AGO2 protein (Fig. 6d). Moreover, ATF7 protein expression was also increased in HCMECs overexpressing circSARS-CV2-N1368 (Fig. 6e). These data indicated that circSARS-CV2-N1368 could increase ATF7 expression by targeting miR-103a-3p in HCMECs. Moreover, ATF7 expression was markedly increased in HCMECs subjected to OGD or TNF-α protein treatment (Supplemental Figs. S10 and S11), indicating that an increase in ATF7 may contribute to EC dysfunction.

miR-103a-3p and siRNA targeting Atf7 (si-Atf7) were separately transfected into HCMECs overexpressing circSARS-CV2-N1368 to confirm whether ATF7 mediates the effect of circSARS-CV2-N1368 on the phenotype of ECs. The results of the Western blot analysis revealed significant increases in VEGFA expression, e-NOS and HH3 activation, and NF-κB p65 inactivation in circSARS-CV2-N1368-overexpressing HCMECs after transfection with miR-103a-3p and si-Atf7 (Fig. 6e).

The elevated ROS level detected in circSARS-CV2-N1368-overexpressing HCMECs was markedly decreased by miR-103a-3p and si-Atf7 (Fig. 6f). Moreover, the results of the EdU assay showed that miR-103a-3p and si-Atf7 reversed the inhibitory effect of circSARS-CV2-N1368 on HCMEC proliferation (Fig. 6g). Consistently, transwell migration and wound healing assays revealed that miR-103a-3p and si-Atf7 could rescue the decrease in the migratory capacity of HCMECs caused by circSARS-CV2-N1368 (Fig. 6h, i). Additionally, miR-103a-3p and si-Atf7 abolished the circSARS-CV2-N1368-mediated inhibition of tube formation in HCMECs (Fig. 6j). Therefore, these results indicate that the miR-103a-3p/ATF7 axis mediates the damaging effect of circSARS-CV2-N1368 on EC function.

### CircSARS-CV2-N1368 increases ATF7 expression to activate TLR4/NF-κB/ROS signaling in ECs

**Fig. 7:**
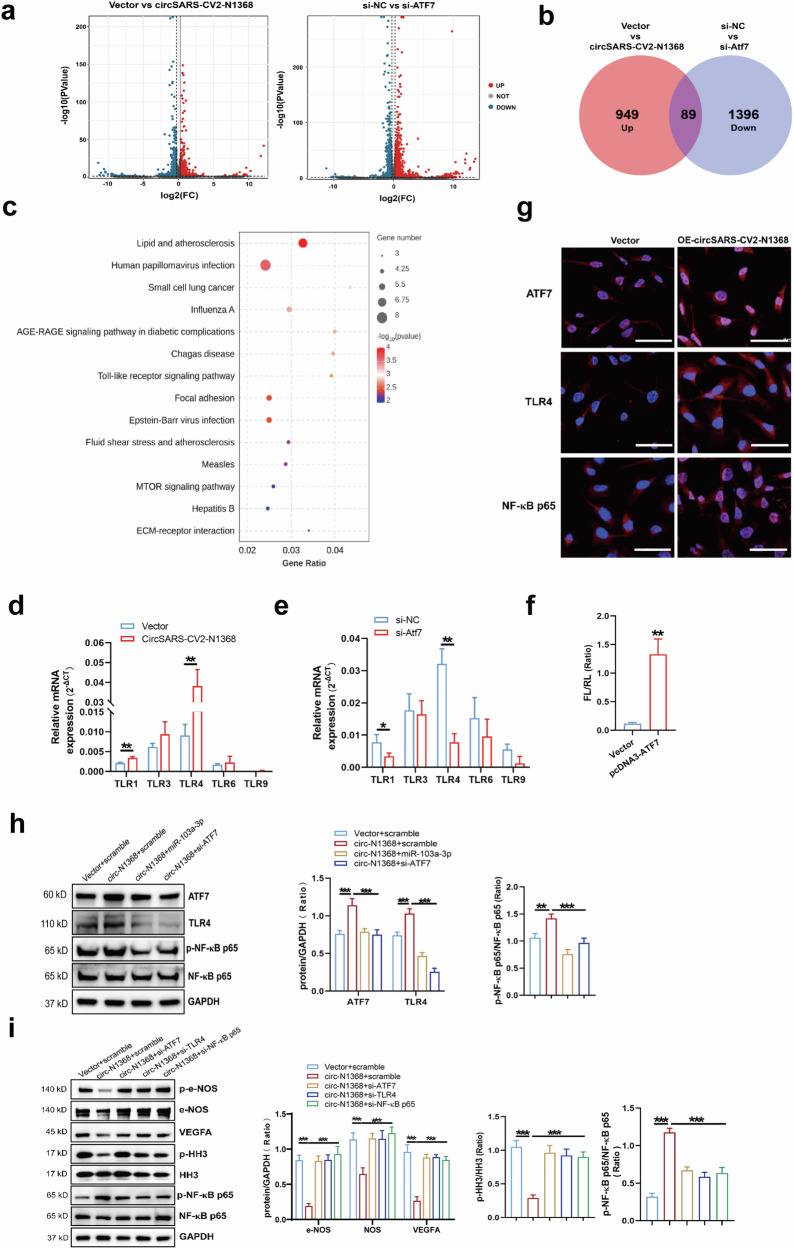

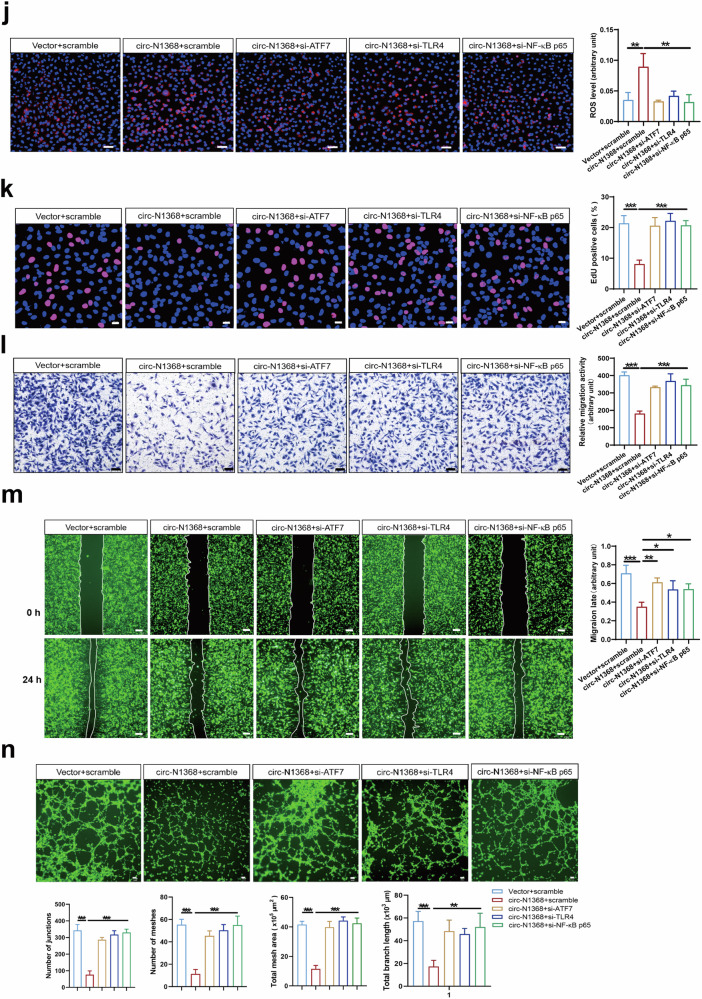
CircSARS-CV2-N1368 regulates the phenotypes of HCMECs by activating TLR4/NF-κB/ROS signal. **a** Volcano plot showing the dysregulated genes in HCMECs by circSARS-CV2-N1368 and ATF7 siRNA, respectively. CircSARS-CV2-N1368-up-regulated genes and ATF7 siRNA-down-regulated genes in HCMECs were overlapped in Venn diagram (**b**) and categorized by KEGG enrichment analysis (**c**). **d**, **e** The expression of Toll-like receptor-related genes in HCMECs with overexpression of circSARS-CV2-N1368 or knock-down of ATF7 by RT-qPCR assay. **f** Transcriptional activation of TLR4 gene by dual luciferase assay. **g** Detection of ATF7, TLR4 and NF-κB p65 in circSARS-CV2-N1368 overexpressing HCMECs with immunofluorescence assay. **h** Expression of ATF7, TLR4 and NF-κB p65 activation in HCMECs by Western blot assay. **i** VEGFA expression, activations of eNOS, HH3 and NF-κB p65 in HCMECs by Western blot assay. **j** Detection of ROS level in HCMECs by using DCFH-DA probe. **k** Proliferation activity of HCMECs by EdU assay. **l**, **m** Migration activity of HCMECs by trans-well migration assay and wound healing assay, respectively. **n** Matrigel tube formation assay of circSARS-CV2-N1368-overexpressiong HCMECs with knock-down of ATF7, TLR4 and NF-κB p65, respectively. Comparisons were made with unpaired *t* test in (**d**)–(**f**), and comparisons were made with 2-way ANOVA in (**h**), (**i**), and 1-way ANOVA in (**j**)–(**n**), *n* = 3 per group. The scale bar is 50 μm in (**g**), (**j**), and (**l**), the scale bar is 100 μm in (**n**), the scale bar is 200 μm in (**m**). *P < 0.05, **P < 0.01, ***P < 0.001.

An RNA sequencing assay was conducted on HCMECs in which circSARS-CV2-N1368 was overexpressed or ATF7 was knocked down to screen the genes downstream of ATF7 (Fig. 7a). Intersectional analyses of the total potential ATF7-modulated genes were performed, and 89 candidate genes were screened (Fig. 7b). Kyoto Encyclopedia of Genes and Genomes enrichment analyses indicated that the Toll-like receptor signaling pathway was regulated by ATF7 in HCMECs (Fig. 7c). The RT‒qPCR results revealed that the Toll-like receptor 4 (TLR4) gene was highly expressed and specifically modulated by ATF7 in HCMECs (Fig. 7d, e). Moreover, the dual-luciferase reporter gene assay confirmed that ATF7 could transcriptionally promote TLR4 expression (Fig. 7f). Immunofluorescence assays revealed that TLR4 and ATF7 expression and the nuclear localization of NF-κB p65 were increased in circSARS-CV2-N1368-overexpressing HCMECs (Fig. 7g). Moreover, the ATF7 and TLR4 mRNAs were upregulated in circSARS-CV2-N1368-overexpressing COs (Supplemental Fig. S12). However, ATF7 knockdown (Supplemental Fig. S13) and the addition of miR-103a-3p attenuated TLR4 expression and NF-κB p65 activation in circSARS-CV2-N1368-overexpressing HCMECs (Fig. 7h). Additionally, miR-103a-3p reversed the increases in ATF7 and TLR4 expression and NF-κB p65 activation in HCMECs exposed to OGD or TNF-α treatment (Supplemental Figs. S14 and S15).

TLR4/NF-κB/ROS signaling is involved in oxidative stress and inflammation in vascular endothelial cells [18–20]. We determined the potential role of the ATF7-TLR4-NF-κB axis in circSARS-CV2-N1368-induced damage to ECs by detecting the effects of knocking down ATF7, TLR4 and NF-κB p65 on the phenotypes of circSARS-CV2-N1368-overexpressing HCMECs. Western blot assays revealed that the absence of ATF7, TLR4 and NF-κB p65 reversed the decrease in VEGFA expression and the inactivation of e-NOS and HH3 in circSARS-CV2-N1368-overexpressing HCMECs (Fig. 7i, Supplemental Fig. S10). The elevated ROS level in circSARS-CV2-N1368-overexpressing HCMECs was markedly decreased in the absence of ATF7, TLR4 or NF-κB p65 (Fig. 7j). Consistently, the results of the EdU assay showed that the knockdown of ATF7, TLR4 and NF-κB p65 reversed the inhibitory effect of circSARS-CV2-N1368 on HCMEC proliferation (Fig. 7k). Transwell migration and wound healing assays revealed that the absence of ATF7, TLR4 and NF-κB p65 reversed the decrease in the migratory capacity of circSARS-CV2-N1368-overexpressing HCMECs (Fig. 7l, m). The circSARS-CV2-N1368-induced suppression of tube formation in HCMECs was also abolished by the knockdown of ATF7, TLR4 and NF-κB p65 (Fig. 7n). Therefore, these data suggest that the ATF7/TLR4/NF-κB axis mediates the circSARS-CV2-N1368-promoted oxidative damage to ECs.

A previous study revealed that the TLR4/NF-κB pathway mediated endotoxin-induced generation of ROS through PKC-activated NAD(P)H oxidase [21]. Consistently, the present study revealed that the knockdown of PKCA, PKCB and CYBB, as well as the NAD(P)H oxidase inhibitors DPI and apocynin, significantly suppressed the increase in ROS levels in circSARS-CV2-N1368-overexpressing HCMECs (Supplemental Figs. S16 and S17). These results indicate that PKC-activated NAD(P)H oxidase also participates in circSARS-CV2-N1368-promoted ROS generation in HCMECs.

### A ROS-scavenging treatment reverses circSARS-CV2-N1368-induced EC dysfunction

**Fig. 8:**
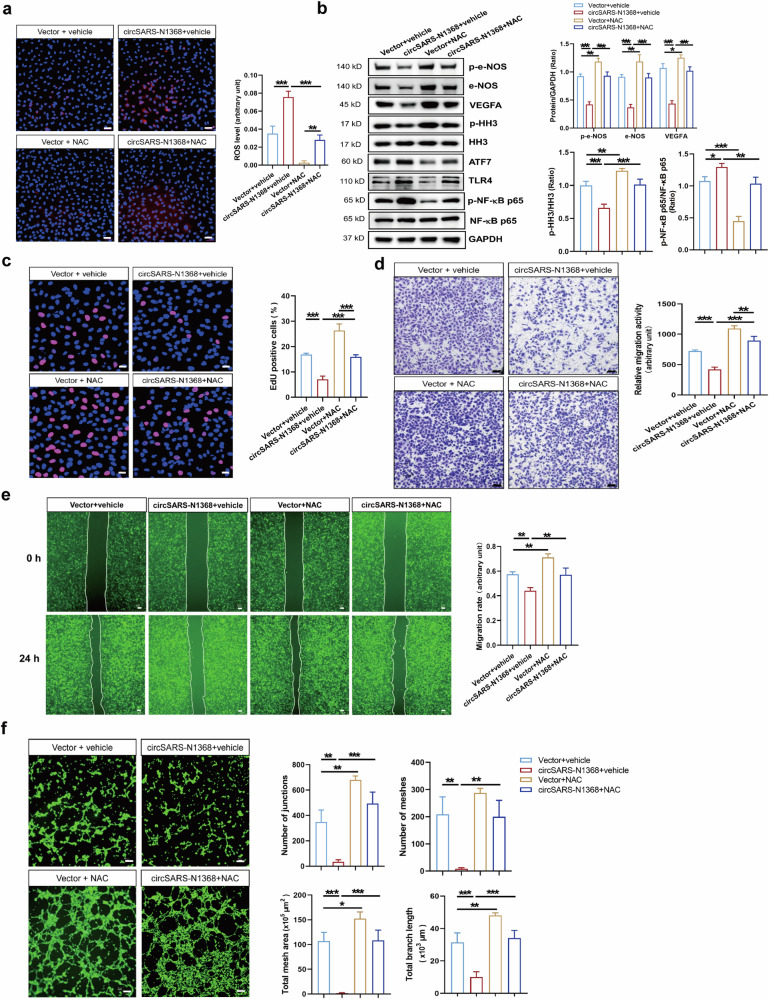
NAC treatment reverses the effect of circSARS-CV2-N1368 on impairment of HCMECs. **a** Detection of ROS level in circSARS-CV2-N1368 overexpressing HCMECs by using DCFH-DA probe. **b** Western blotting analysis of VEGFA expression, activations of e-NOS and HH3 in NAC-treated HCMECs with overexpression of circSARS-CV2-N1368. **c** Proliferation activity analysis of HCMECs by EdU assay. **d**, **e** Migration activity analysis of HCMECs was evaluated by trans-well migration assay and wound healing assay, respectively. **f** NAC rescued matrigel tube formation of HCMECs with overexpression of circSARS-CV2-N1368. Comparisons were made with 1-way ANOVA, *n* = 3 per group in (**a**)–(**f**), and 2-way ANOVA, *n* = 3 per group in (**b**), The scale bar is 50 μm in (**a**), (**c**), (**d**), the scale bar is 100 μm in (**f**), the scale bar is 200 μm in (**e**). *P < 0.05, **P < 0.01, ***P < 0.001.

ROS-induced oxidative stress is an important mechanism underlying endothelial cell dysfunction [22]. The ROS scavenger NAC was used to decrease ROS levels in HCMECs and to elucidate the role of ROS in the circSARS-CV2-N1368-induced impairment of HCMECs (Fig. 8a). The Western blot results showed that the inactivation of e-NOS and HH3 and the decrease in VEGFA levels were reversed by NAC in circSARS-CV2-N1368-overexpressing HCMECs. Moreover, circSARS-CV2-N1368-induced ATF7 and TLR4 expression and NF-κB activation were attenuated by NAC treatment (Fig. 8b). The results of the EdU assay revealed that NAC mitigated the inhibitory effect of circSARS-CV2-N1368 on HCMEC proliferation (Fig. 8c). Consistently, the results of the transwell migration and wound healing assays indicated that NAC reversed the reduced migratory activity of circSARS-CV2-N1368-overexpressing HCMECs (Fig. 8d, e). Moreover, NAC treatment reversed the decrease in the tube formation ability of HCMECs caused by circSARS-CV2-N1368 (Fig. 8f). Together, these results demonstrate that ROS scavenging can ameliorate circSARS-CV2-N1368-induced EC dysfunction.

## Discussion

A previous report revealed that circRNAs are highly expressed from the region from the ORF1ab to the ORF8/N gene of SARS-CoV-2 [9]. The nucleocapsid protein of SARS-CoV-2 is known as an abundant and highly immunogenic protein in coronaviruses with a highly conserved gene sequence [23, 24]; however, whether circRNAs derived from the N gene participate in SARS-CoV-2-induced cardiovascular sequelae remains unexplored. Our present study identified 3 novel circRNAs derived from the SARS-CoV-2 N gene. Notably, circSARS-CV2-N1368 inhibited HCMEC proliferation and migration and angiogenesis in vitro and in vivo and inhibited EC-dependent vascular relaxation and CO beating, indicating that circSARS-CV2-N1368 may play an important role in initiating EC dysfunction.

Thrombosis in venous and arterial circulatory beds is a prominent feature of SARS-CoV-2 infection [25, 26]. The inflammation of ECs contributes to the degranulation and exocytosis of Weibel–Palade bodies (WPBs), which promote the recruitment of platelets [27]. In the present study, for the first time, we identified that platelets could be markedly recruited to HCMECs with exogenous overexpression of circSARS-CV2-N654, -N804, or -N1368. Moreover, the expression of inflammatory genes, including IL-1B, IL-6, TNF, VCAM-1, and ICAM-1, was upregulated in HCMECs by these three circRNAs. Furthermore, NF-κB activation was confirmed in HCMECs with exogenous overexpression of circSARS-CV2-N1368. These results suggest that circSARS-CV2-N654, -N804, and -N1368 may activate ECs to recruit platelets, contributing to thrombosis in blood vessels.

Hypertension has been identified as the most prevalent cardiovascular comorbidity in COVID-19 patients and demonstrably increases the risks of hospitalization and death [28, 29]. Recent studies have shown increases in systolic and diastolic blood pressure or an increased proportion of hypertensive patients during the COVID-19 pandemic [30–32]. In this study, circSARS-CV2-N1368 was shown to impair the EC-dependent diastolic response of the coronary artery by inhibiting eNOS expression and activation in ECs, suggesting that circSARS-CV2-N1368 may contribute to the occurrence of hypertension in COVID-19 patients.

Patients who simultaneously suffer acute coronary syndrome (ACS) and COVID-19 may exhibit worse clinical outcomes [33]. Endothelial damage in COVID-19-positive ACS patients may be caused by direct infiltration of the endothelium by SARS-CoV-2, resulting in endothelial dysfunction [34–36]. In the present study, exogenous overexpression of circSARS-CV2-N1368 increased ROS levels, decreased VEGFA and eNOS expression in ECs, inhibited EC proliferation and migration, and markedly suppressed angiogenesis in vitro and in vivo. These data indicate that circSARS-CV2-N1368 promotes EC dysfunction and may contribute to SARS-CoV-2-induced endothelial injury.

Cardiomyocytes, fibroblasts and ECs are fundamental cell types in myocardial tissue, and previous reports have shown that SARS-CoV-2 binds to ACE2 in cardiomyocytes, leading to cell death and the expression of inflammatory factors [37]. Moreover, SARS-CoV-2 has the potential to induce ACE2 expression in fibroblasts and exert pathological effects on fibroblasts [37]. Because of the low level of ACE2 in ECs, although COVID-19 patients exhibit evident endothelial dysfunction [38], ECs can be infected only when they are exposed to very high concentrations of SARS-CoV-2 [39, 40], and SARS-CoV-2-induced endothelial injury may be secondary to the infection of adjacent cells or activation of immune cells, platelets, and proinflammatory cytokines [41]. Noncoding RNAs, such as miRNAs and circRNAs, can be secreted from cells through cell particles [42]. In this study, circSARS-CV2-N654, -N608, -N750, and -N1368 were detected in the blood serum of hACE2 transgenic mice with SARS-CoV-2 infection, indicating that SARS-CoV-2 circRNAs may be delivered into myocardial tissue and the vascular endothelium via the peripheral blood circulation.

In the present study, the effects of circSARS-CV2-Ns on the phenotypes of cardiomyocytes and fibroblasts were also explored, and no significant effects were observed on neonatal mouse cardiomyocytes or fibroblasts with exogenous expression of circSARS-CV2-N654, -N804, -N1368. Moreover, circSARS-CV2-N654, -N804, and -N1368 were also overexpressed in COs, and the mRNA expression of eNOS and VEGFA was markedly decreased by circSARS-CV2-N1368, which is consistent with the inhibitory effect of circSARS-CV2-N1368 on eNOS and VEGFA expression in HCMECs. These findings suggest that circSARS-CV2-N1368 may mainly impair endothelial function.

Our study showed that circSARS-CV2-N1368 specifically binds to miR-103a-3p to upregulate ATF7 expression, and miR-103a-3p overexpression or ATF7 deficiency was proven to reverse the circSARS-CV2-N1368-induced EC impairment. Moreover, miR-103a-3p is highly expressed in the brain, heart, arteries and veins (https://ccb-web.cs.uni-saarland.de/tissueatlas2/patterns.), and it was also reported to promote SARS-CoV-2 RNA degradation by targeting the 3’ UTR of the viral genomic RNA [43]. ATF7 is a transcription factor that interacts with JDP2 to activate inflammatory gene expression [44]. Consistent with these findings, we revealed that ATF7 knockdown could ameliorate circSARS-CV2-N1368-promoted NF-κB activation and ROS generation. Additionally, our study identified TLR4 as a downstream gene of ATF7, and ATF7 could transcriptionally promote TLR4 expression to activate NF-κB signaling and ROS production in ECs, contributing to circSARS-CV2-N1368-induced oxidative damage in ECs. Since TLR4/NF-κB signaling plays a key role in the inflammatory response [45], our study also revealed that TLR4/NF-κB mediated ROS production in ECs [20]. Therefore, the results of the present study showed that circSARS-CV2-N1368 impairs ECs by sponging miR-103a-3p to increase ATF7 expression and activate TLR4/NF-κB/ROS signaling.

ROS and reactive nitrogen species (RNS) production might be activated either by viral components or by cytokines secreted in response to the pathogen, which play important roles in the innate antiviral immune response. However, they might have a deleterious effect on coronavirus infection [46], and antioxidants may be potential therapeutic tools for COVID-19 patients [47]. In addition to the mitochondrial source, ROS are produced by the NADPH oxidase Nox2 during SARS-CoV-2 infection [48]. Our study revealed that circSARS-CV2-N654, -N804, and -N1368 could increase ROS production in ECs and that ATF7/TLR4/NF-κB signaling and PKC-activated NAD(P)H oxidase mediated circSARS-CV2-N1368-induced ROS generation in ECs. Importantly, the ROS scavenger NAC reversed the circSARS-CV2-N1368-induced impairment of EC function. Intriguingly, our study revealed that NAC mitigated ATF7/TLR4/NF-κB activation, suggesting that circSARS-CV2-N1368 promotes a positive feedback loop of ATF7/TLR4/NF-κB/ROS signaling in ECs.

Plasma samples from COVID-19 patients have been very difficult to obtain during the COVID-19 pandemic. In this study, we detected the levels of 5 circSARS-CV2-Ns in pharyngeal swab samples and the peripheral blood serum of hACE2 transgenic mice infected with SARS-CoV-2 but were not able to determine the circSARS-CV2-N and miR-103a-3p levels in the peripheral blood of COVID-19 patients. In the present study, downregulated VEGFA and eNOS expression levels were observed in circSARS-CV2-N1368-overexpressing rat coronary arteries and HCMECs, and whether the downregulation of these two genes was directly modulated by circSARS-CV2-N1368 or was the consequence of circSARS-CV2-N1368-promoted ROS production warrants further investigation.

**Fig. 9:**
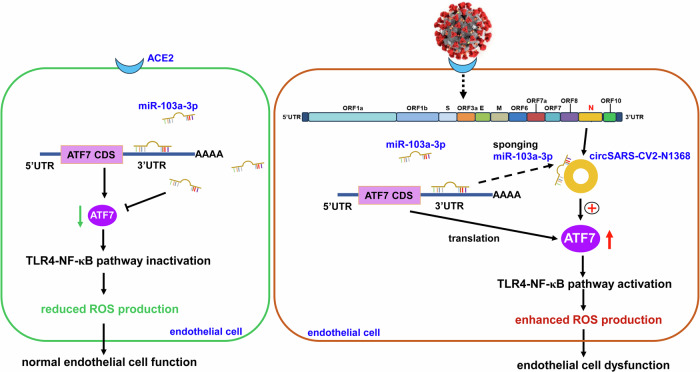
Working model of circSARS-CV2-N1368 impairing endothelial cell function through ATF7/TLR4/NF-κB/ROS pathway. In normal ECs, miR-103a-3p inhibits TLR4/NF-κB signal to attenuate ROS production by targeting ATF7. During SARS-CoV-2 infection, exogenous circSARS-CV2-N1368 sponges miR-103a-3p to upregulate ATF7 and activate TLR4/NF-κB/ROS signal to augment ROS production and oxidative damage in ECs, contributing to circSARS-CV2-N1368-promoted ECs dysfunction.

In summary, our study identified the novel circSARS-CV2-N654, -N804, -N1368 encoded by SARS-CoV-2. Exogenous overexpression of these circRNAs could enhance the ability of ECs to adhere to platelets; increase ROS production; and inhibit EC proliferation, migration and angiogenesis. Importantly, our study showed that circSARS-CV2-N1368 sponges miR-103a-3p to activate ATF7/TLR4/NF-κB signaling and aggravate ROS production and EC dysfunction (Fig. 9). Therefore, circSARS-CV2-N1368 and miR-103a-3p may be potential candidates for novel nucleic acid drugs to treat COVID-19.

## Supplementary information

Supplementary data
