# Supplementary material for: CircSARS-CV2-N1368 from SARS-CoV-2 impairs endothelial cell function through the upregulation of ATF7 to activate TLR4/NF-κB/ROS signaling
Source: Acta Pharmacol Sin. 2025 Mar 11;46(8):2180–95. doi: 10.1038/s41401-025-01516-8 (PMC12274418; doi:10.1038/s41401-025-01516-8)
Supplement: Supplementary file 1 — Supplementary data [file 41401_2025_1516_MOESM1_ESM.pdf]

Supplementary data

circSARS-CV2-N608 sequence：
acccaataatactgcgtcttggttcaccgctctcactcaacatggcaaggaagaccttaaattccctcgaggacaaggcg
ttccaattaacaccaatagcagtccagatgaccaaattggctactaccgaagagctaccagacgaattcgtggtggtgac
ggtaaaatgaaagatctcagtccaagatggtatttctactacctaggaactgggccagaagctggacttccctatggtgc
taacaaagacggcatcatatgggttgcaactgagggagccttgaatacaccaaaagatcacattggcacccgcaatcctg
ctaacaatgctgcaatcgtgctacaacttcctcaaggaacaacattgccaaaaggcttctacgcagaagggagcagaggc
ggcagtcaagcctcttctcgttcctcatcacgtagtcgcaacagttcaagaaattcaactccaggcagcagtaggggaac
ttctcctgctagaatggctggcaatggcggtgatgctgctcttgctttgctgctgcttgacagattgaaccagcttgaga
gcaaaatgtctggtaaaggccaacaacaacaaggccaaactgtcacta

circSARS-CV2-N654 sequence：
gagaacgcagtggggcgcgatcaaaacaacgtcggccccaaggtttacccaataatactgcgtcttggttcaccgctctc
actcaacatggcaaggaagaccttaaattccctcgaggacaaggcgttccaattaacaccaatagcagtccagatgacca
aattggctactaccgaagagctaccagacgaattcgtggtggtgacggtaaaatgaaagatctcagtccaagatggtatt
tctactacctaggaactgggccagaagctggacttccctatggtgctaacaaagacggcatcatatgggttgcaactgag
ggagccttgaatacaccaaaagatcacattggcacccgcaatcctgctaacaatgctgcaatcgtgctacaacttcctca
aggaacaacattgccaaaaggcttctacgcagaagggagcagaggcggcagtcaagcctcttctcgttcctcatcacgta
gtcgcaacagttcaagaaattcaactccaggcagcagtaggggaacttctcctgctagaatggctggcaatggcggtgat
gctgctcttgctttgctgctgcttgacagattgaaccagcttgagagcaaaatgtctggtaaaggccaacaacaacaagg
ccaaactgtcacta

circSARS-CV2-N750 sequence：
atactgcgtcttggttcaccgctctcactcaacatggcaaggaagaccttaaattccctcgaggacaaggcgttccaatt
aacaccaatagcagtccagatgaccaaattggctactaccgaagagctaccagacgaattcgtggtggtgacggtaaaat
gaaagatctcagtccaagatggtatttctactacctaggaactgggccagaagctggacttccctatggtgctaacaaag
acggcatcatatgggttgcaactgagggagccttgaatacaccaaaagatcacattggcacccgcaatcctgctaacaat
gctgcaatcgtgctacaacttcctcaaggaacaacattgccaaaaggcttctacgcagaagggagcagaggcggcagtca
agcctcttctcgttcctcatcacgtagtcgcaacagttcaagaaattcaactccaggcagcagtaggggaacttctcctg
ctagaatggctggcaatggcggtgatgctgctcttgctttgctgctgcttgacagattgaaccagcttgagagcaaaatg
tctggtaaaggccaacaacaacaaggccaaactgtcactaagaaatctgctgctgaggcttctaagaagcctcggcaaaa
acgtactgccactaaagcatacaatgtaacacaagctttcggcagacgtggtccagaacaaacccaaggaaattttgggg
accaggaactaatcagacaaggaactgatt

circSARS-CV2-N804 sequence：
acgtttggtggaccctcagattcaactggcagtaaccagaatggagaacgcagtggggcgcgatcaaaacaacgtcggcc
ccaaggtttacccaataatactgcgtcttggttcaccgctctcactcaacatggcaaggaagaccttaaattccctcgag
gacaaggcgttccaattaacaccaatagcagtccagatgaccaaattggctactaccgaagagctaccagacgaattcgt
ggtggtgacggtaaaatgaaagatctcagtccaagatggtatttctactacctaggaactgggccagaagctggacttcc
ctatggtgctaacaaagacggcatcatatgggttgcaactgagggagccttgaatacaccaaaagatcacattggcaccc
gcaatcctgctaacaatgctgcaatcgtgctacaacttcctcaaggaacaacattgccaaaaggcttctacgcagaaggg
agcagaggcggcagtcaagcctcttctcgttcctcatcacgtagtcgcaacagttcaagaaattcaactccaggcagcag

taggggaacttctcctgctagaatggctggcaatggcggtgatgctgctcttgctttgctgctgcttgacagattgaacc
agcttgagagcaaaatgtctggtaaaggccaacaacaacaaggccaaactgtcactaagaaatctgctgctgaggcttct
aagaagcctcggcaaaaacgtactgccactaaagcatacaatgtaacacaagctttcggcagacgtggtccagaacaaac
ccaa

circSARS-CV2-N1368 sequence：
ttagcctttctgctattccttgttttaattatgcttattatcttttggttctcacttgaactgcaagatcataatgaaac
ttgtcacgcctaaacgaacatgaaatttcttgttttcttaggaatcatcacaactgtagctgcatttcaccaagaatgta
gtttacagtcatgtactcaacatcaaccatatgtagttgatgacccgtgtcctattcacttctattctaaatggtatatt
agagtaggagctagaaaatcagcacctttaattgaattgtgcgtggatgaggctggttctaaatcacccattcagtacat
cgatatcggtaattatacagtttcctgtttaccttttacaattaattgccaggaacctaaattgggtagtcttgtagtgc
gttgttcgttctatgaagactttttagagtatcatgacgttcgtgttgttttagatttcatctaaacgaacaaactaaaa
tgtctgataatggaccccaaaatcagcgaaatgcaccccgcattacgtttggtggaccctcagattcaactggcagtaac
cagaatggagaacgcagtggggcgcgatcaaaacaacgtcggccccaaggtttacccaataatactgcgtcttggttcac
cgctctcactcaacatggcaaggaagaccttaaattccctcgaggacaaggcgttccaattaacaccaatagcagtccag
atgaccaaattggctactaccgaagagctaccagacgaattcgtggtggtgacggtaaaatgaaagatctcagtccaaga
tggtatttctactacctaggaactgggccagaagctggacttccctatggtgctaacaaagacggcatcatatgggttgc
aactgagggagccttgaatacaccaaaagatcacattggcacccgcaatcctgctaacaatgctgcaatcgtgctacaac
ttcctcaaggaacaacattgccaaaaggcttctacgcagaagggagcagaggcggcagtcaagcctcttctcgttcctca
tcacgtagtcgcaacagttcaagaaattcaactccaggcagcagtaggggaacttctcctgctagaatggctggcaatgg
cggtgatgctgctcttgctttgctgctgcttgacagattgaaccagcttgagagcaaaatgtctggtaaaggccaacaac
aacaaggccaaactgtcactaagaaatctgctgctgaggcttctaagaagcctcggcaaaaacgtactgccactaaagca
tacaatgtaacacaagctttcggcagacgtggtccagaacaaacccaaggaaattttggggaccaggaactaatcagaca
aggaactg

Fig. 1. The nucleotide sequence of five circRNAs derived from SARS-CoV-2 N gene.

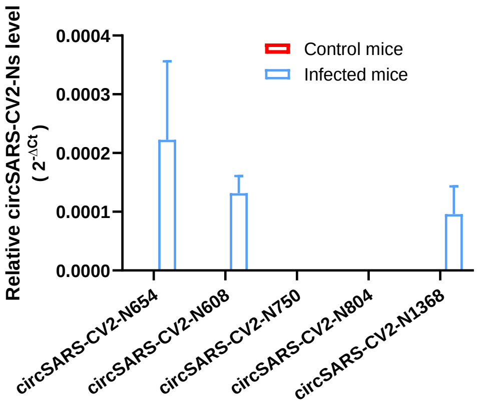

Fig. 2. Levels of circSARS-CV2-N654, -N608, -N750, -N804, and -N1368 in the peripheral blood serum of humanized ACE2 transgenic mice with SARS-CoV-2 infection. Blood serum from 5 hACE2 transgenic mice received SARS-CoV-2 infection and 5 control mice was detected by RT-qPCR assay, none of the above circRNAs could be detected in control mice.

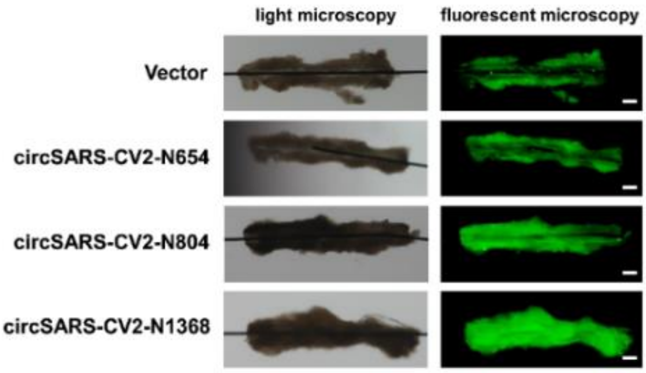

Fig. 3. Preparation of rat coronary arteries with overexpression of circSARS-CV2-Ns for endothelium-dependent vasorelaxation assay. Co-expression of green fluorescence protein (GFP) indicated that rat coronary arteries were efficiently infected by circSARS-CV2-N654, -N804, -N1368, respectively. The scale bar is 200 μm.

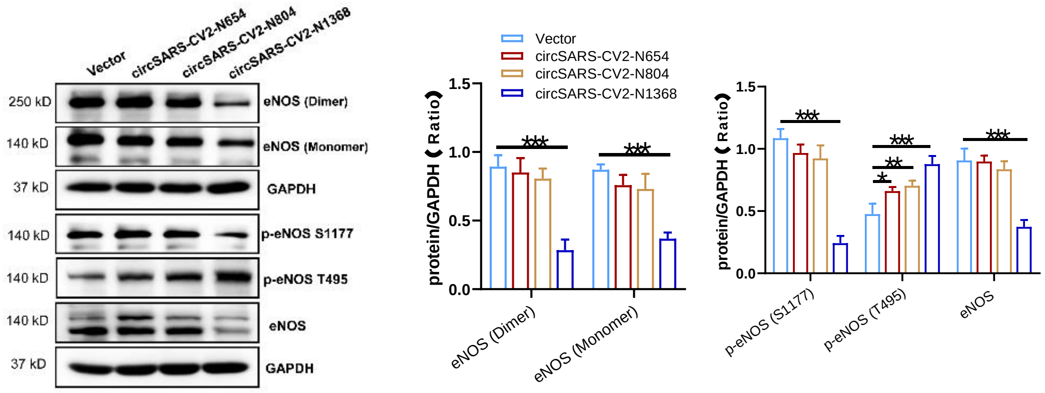

Fig. 4. Overexpression of circSARS-CV2-N1368 inactivate eNOS in HCMECs. Levels of total eNOS and the active phosphorylated eNOS (S1177) level were decreased, accompanied by the reduced eNOS dimer, while the inactive phosphorylated eNOS (T495) level was elevated in circSARS-CV2-N1368 overexpressing HCMECs. Comparisons were made with 2-way ANOVA, n=3 per group. *P<0.05, **P<0.01, ***P<0.001.

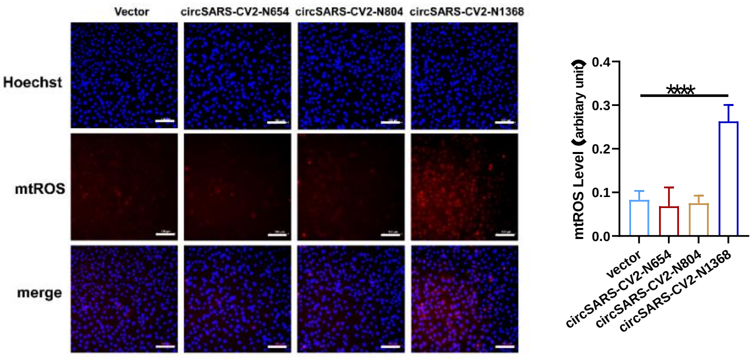

Fig. 5. Overexpression of circSARS-CV2-N1368 enhances mitochondrial ROS in HCMECs. Level of mitochondrial ROS in HCMECs was detected by using MitoROS Red probe. Comparisons were made with one-way ANOVA, n=3 per group. ****P<0.0001.

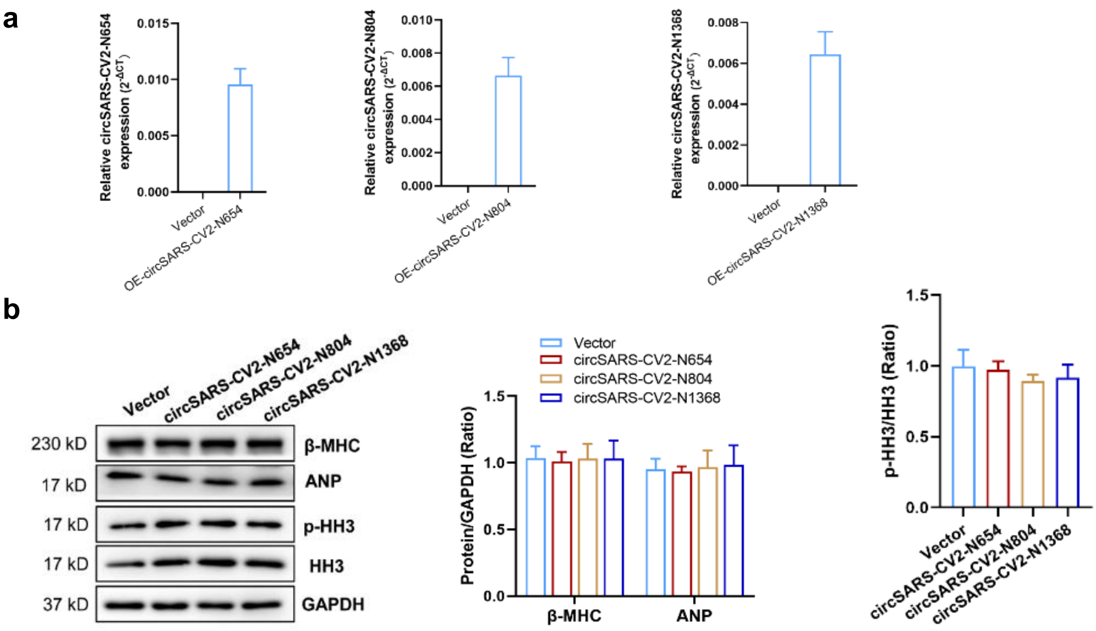

Fig. 6. Effects of circSARS-CV2-Ns overexpression on hypertrophy-related gene expression and HH3 activation in neonatal mouse ventricular cardiomyocytes (NMVCs). **a** Detection of exogenous overexpression SARS-CV2-Ns in NMVCs by RT-qPCR assay. **b** No significant differences in β-MHC, ANP expression and HH3 activation in NMVCs with overexpression of circSARS-CV2-N654, -N804, -N1368, respectively (1-way ANOVA, 2-way ANOVA, n=3 per group).

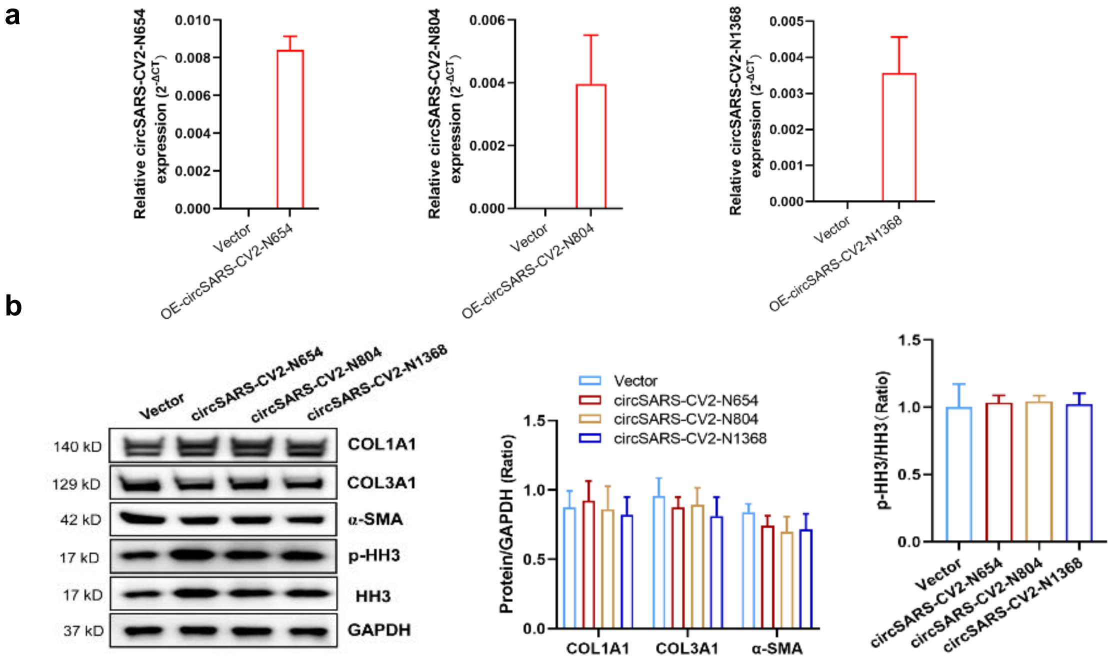

Fig. 7. Effects of circSARS-CV2-Ns overexpression on fibrosis-related gene expression and HH3 activation in mouse cardiac fibroblasts (mCFs). **a** Detection of exogenous overexpression SARS-CV2-Ns in mCFs by RT-qPCR assay. **b** No significant differences in COL1A1, COL3A1 and α-SMA expression and HH3 activation in mCFs with overexpression of circSARS-CV2-N654, -N804, -N1368, respectively (1-way ANOVA, 2-way ANOVA, n=3 per group).

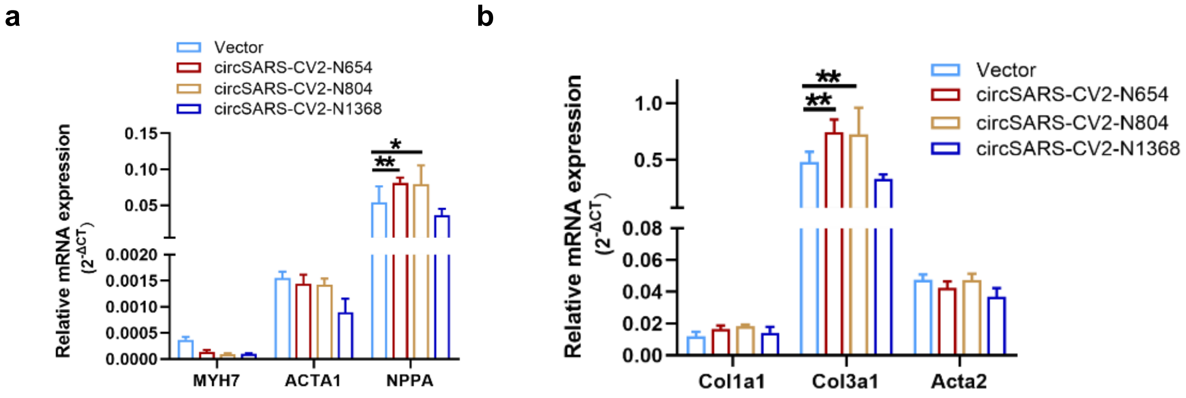

Fig. 8. Effects of circSARS-CV2-Ns overexpression on cardiac hypertrophy and fibrosis-related gene expression in cardiac organoids. **a** Detection of cardiac hypertrophy-related gene expression by RT-qPCR assay. **b** Detection of cardiac fibrosis-related gene expression by RT-qPCR assay (2-way ANOVA, n=3 per group). *$p < 0.05$, **$p < 0.01$.

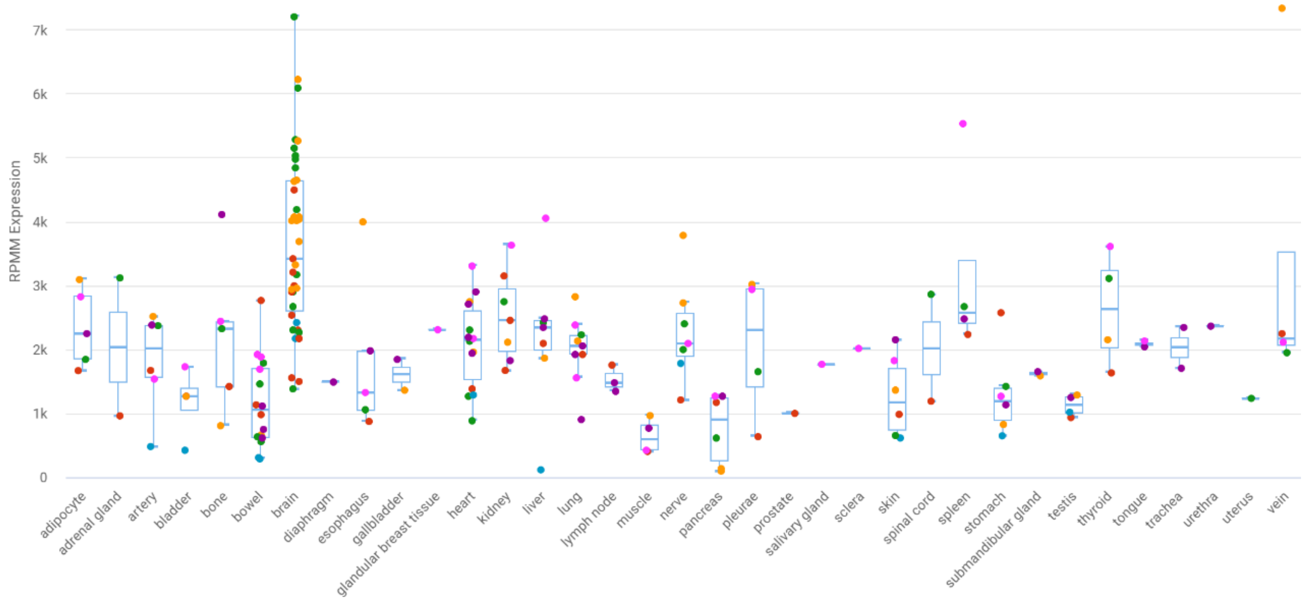

Fig. 9. Expression of miR-103a-3p in human tissues. miR-103a-3p is highly expressed in human brain, artery, vein and heart. Data are from https://ccb-web.cs.uni-saarland.de/tissueatlas2/patterns

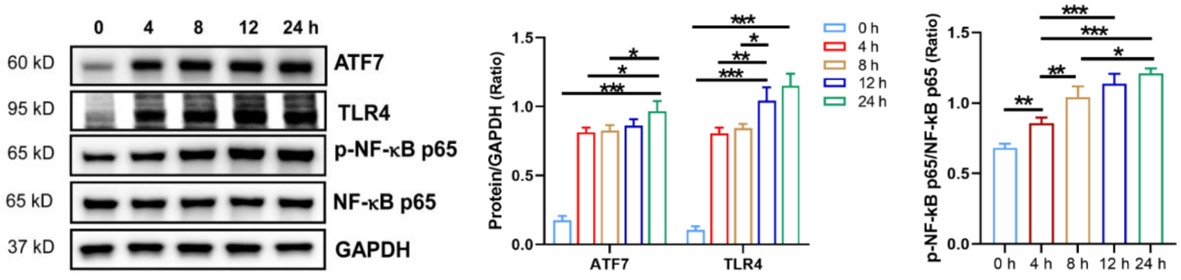

Fig. 10. Oxygen and glucose deprivation (OGD) enhanced ATF7, TLR4 expression and NF-κB p65 activation in human cardiac microvascular endothelial cells (HCMECs). ATF7, TLR4 expression, NF-κB p65 activation in OGD-induced HCMECs by Western blot assay. Comparisons were made with 1-way ANOVA and 2-way ANOVA, n=3 per group. *P<0.05, **P<0.01, ***P<0.001.

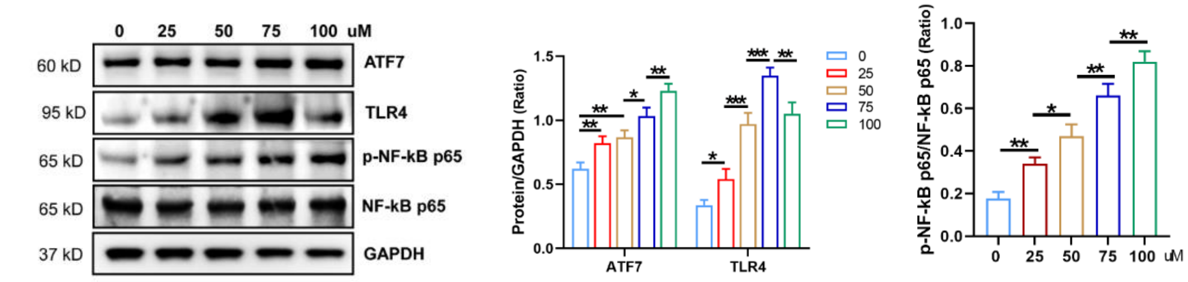

Fig. 11. TNF-α treatment enhanced ATF7, TLR4 expression and NF-κB p65 activation in HCMECs. ATF7, TLR4 expression, NF-κB p65 activation in TNF-α-treated HCMECs by Western blot assay. Comparisons were made with 1-way ANOVA and 2-way ANOVA, *n*=3 per group. **P*<0.05, ***P*<0.01, ****P*<0.001.

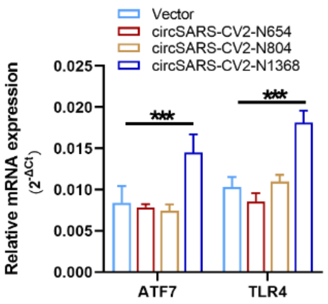

Fig. 12. Upregulations of ATF7 and TLR4 in circSARS-CV2-N1368-overexpressing cardiac organoids. ATF7 and TLR4 mRNA expression in cardiac organoids by RT-qPCR assay. Comparisons were made with 1-way ANOVA, n=3 per group. ****P*<0.001.

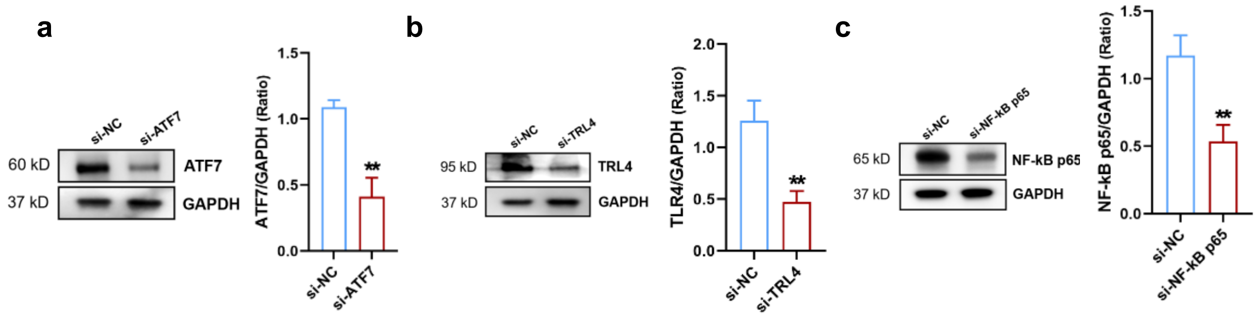

Fig. 13. Small interfering RNA(siRNA)-mediated knock-down of ATF7, TLR4 and NF-κB p65 in HCMECs. ATF7, TLR4 and NF-κB p65 expression in HCMECs by Western blot assay. Comparisons were made with unpaired *t* test in **a**, **b, c**, n=3 per group. ***P*<0.01.

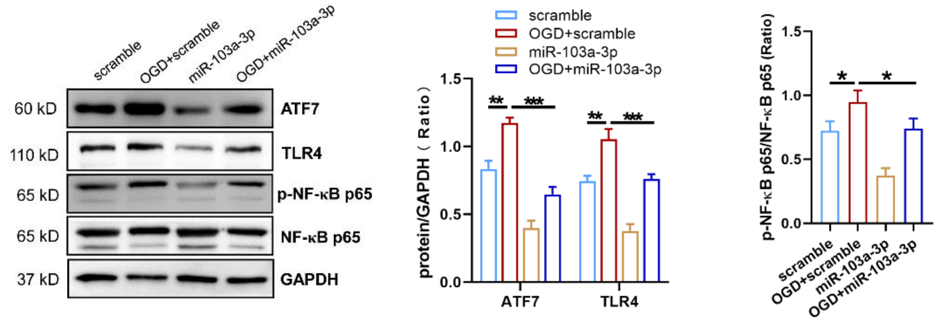

Fig. 14. MiR-103a-3p reversed ATF7, TLR4 expression and NF-κB p65 activation in OGD-treated HCMECs. ATF7, TLR4 expression, NF-κB p65 activation in OGD-treated HCMECs by Western blot assay. Comparisons were made with 1-way ANOVA and 2-way ANOVA, n=3 per group. *P<0.05, ***P<0.001.

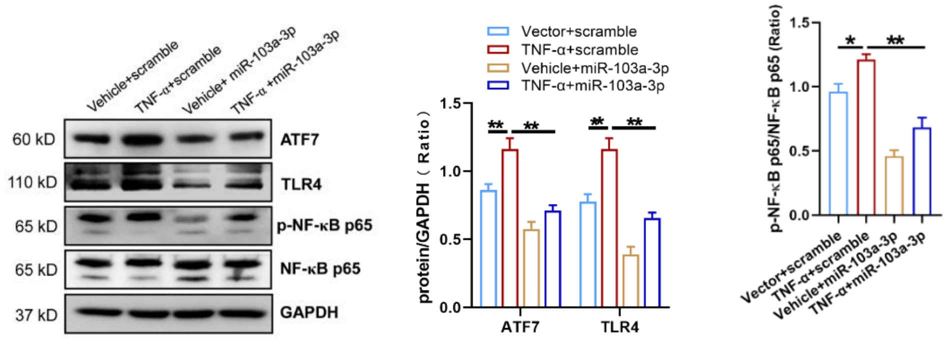

Fig. 15. MiR-103a-3p reversed ATF7, TLR4 expression and NF-κB p65 activation in TNF-α-treated HCMECs. ATF7, TLR4 expression, NF-κB p65 activation in TNF-α-treated HCMECs by Western blot assay. Comparisons were made with 1-way ANOVA and 2-way ANOVA, n=3 per group. **P<0.01, ***P<0.001.

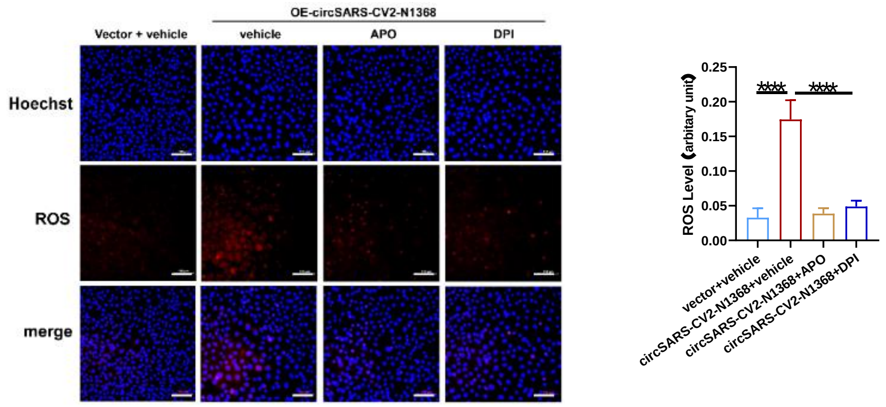

Fig. 16. CircSARS-CV2-N1368-induced ROS can be abolished in HCMECs exposed to NAD(P)H oxidase inhibitors Apocynin (5 mM) and DPI (25 uM) treatment. ROS level in HCMECs was detected by using DCFH-DA probe. Comparisons were made with one-way ANOVA, *n*=3 per group. ****P<0.0001.

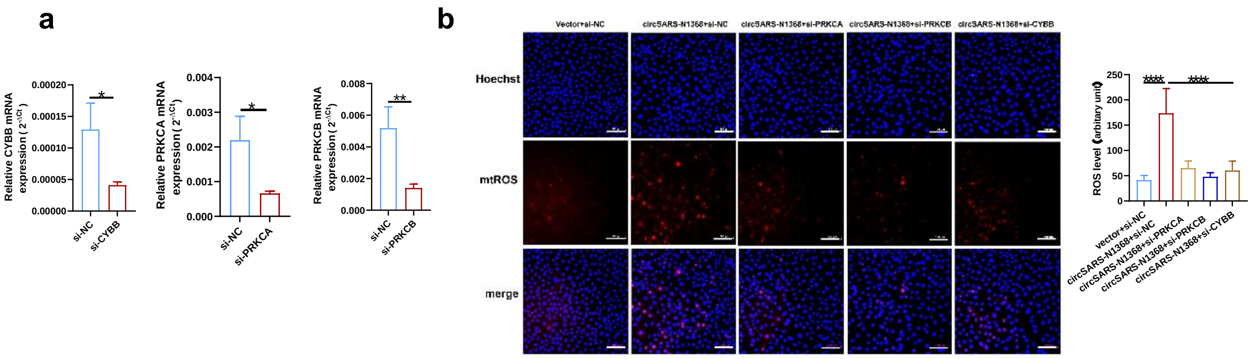

Fig. 17. CircSARS-CV2-N1368-induced ROS can be abolished in HCMECs with knockdown of Nox2, PKCA and PKCB, respectively. **a** MRNA expression of CYBB, PRKCA and PRKCB in HCMECs by RT-qPCR assay. Comparisons were made with unpaired *t* test, n=3 per group. *P<0.05, **P<0.01. **b** ROS level in HCMECs was detected by using DCFH-DA probe. Comparisons were made with one-way ANOVA, *n*=3 per group. ****P<0.0001.

Table 1. Antibodies, reagents and resources

REAGENT or RESOURCE	SOURCE	IDENTIFIER	DILUTION
**Antibodies used for Western blotting**			
Anti-p-eNOS (phospho Ser1177)	Immunoway	YPO514	1:1000
Anti-p-eNOS (phospho Thr495)	Singalway Antibody	#11711	1:500
Anti-eNOS	Abcam	ab199956	1:1000
Anti-VEGFA	Abcam	ab214424	1:1000
Anti-ATF7	Proteintech	29770-1-AP	1:1000
Anti-p-NF-κB p65	Proteintech	48498	1:1000
Anti-NF-κB p65	Proteintech	8242S	1:1000

Anti-p-HH3	CST	53348T	1:1000
Anti-HH3	CST	4620S	1:1000
Anti-TLR4	Proteintech	19811-1-AP	1:1000
Anti-GAPDH	Proteintech	60004	1:5000
**First antibodies used for Immunofluorescence**			
Anti-ATF7	Proteintech	29770-1-AP	1:500
Anti-TLR4	Proteintech	19811-1-AP	1:200
Anti-NF-κB p65	Proteintech	48498	1:500
**Chemicals**			
DAPI(4,6-Diamidino-2-Phenylindole,Dihydrochloride)	SIGMA	F6057	
MitoROS Red	Medchem Express	309704	
Apocynin(4'-Hydroxy-3'-methoxy-acetophenone)	SIGMA	A10809-25G	
Diphenyleneiodonium chloride	SIGMA	D2926-10MG	
Hoechst 33342	Ribobio	C10310-1	
EDU	Ribobio	C10310-1	
TRIzol Reagant (RNAiso Pus)	TAKARA	AG21102	
Lipofectamine^{TM} RNAi MAX	Invitrogen	2840622	
5×RT Primer Mix	TAKARA	AG11706	
2×SYBR	TAKARA	AG11701	
Matrigel Gel	ABW	827045	
Reactive Oxygen Species Assay Kit	Solarbio	CA1420	
RIP Assay	BersinBio	Bes5101(N)	
0.25 % trypsin	Gibco	2756241	
DMEM	Gibco	6124526	
Bovine Serum Albumin	ZETA	Z7010FBS-500	
**Experimental animals and cell**			
C57BL/6（3-4 weeks）	Guangdong Provincial Medical Laboratory Animal Center		
HCMECs	PriMed Cell Technology Company	PriMed-iCell-002	
**Software and Algorith**			
Image J	NIH	https://imagej.net/software/imagej/	
Photoshop CS	Adobe	https://www.adobe.com	
GraphPad Prism	GraphPad	https://www.graphpad.com/	

Table 2. Sequence of PCR primers

Gene	The primer sequence (5′- 3′)	Product (bp)
CircSARS-CV2-N608	F: GCTGCTGCTTGACAGATTGA	226
	R: TTCGTCTGGTAGCTCTTCGGT	
CircSARS-CV2-N654	F: GCTGCTTGACAGATTGAACC	236
	R: GGTCATCTGGACTGCTATTG	
CircSARS-CV2-N750	F: TAAAGCATACAATGTAACACAAG	235
	R: TCGTCTGGTAGCTCTTCGG	
CircSARS-CV2-N804	F: AATCTGCTGCTGAGGCTTCT	216
	R: ACCAAGACGCAGTATTATTGG	
CircSARS-CV2-N1368	F: AACACAAGCTTTCGGCAGAC	250
	R: GTTCGTTTAGGCGTGACAAGT	
eNOS	F: GTGAGACCTTCTGTGTGGGA	208
	R: CTCGTGGACTTGCTGCTTTG	
VEGFA	F: CGGTATAAGTCCTGGAGCGT	188
	R: TTTAACTCAAGCTGCCTCGC	
IL-1B	F: CGAATCTCCGACCACCACTA	186
	R: AGCCTCGTTATCCCATGTGT	
IL-6	F: CAGCCACTCACCTCTTCAGA	206
	R: ACCAGGCAAGTCTCCTCATT	
TNF	F: CCTCAGCCTCTTCTCCTTCC	191
	R: GGTTTGCTACAACATGGGCT	
VCAM-1	F: AAGGTTCCTAGCGTGTACCC	225
	R：CTGCCTTTGTTTGGGTTCGA	
ICAM-1	F: TGATGGGCAGTCAACAGCTA	246
	R: TGATCTCTCCTCACCAGCAC	
MYH7	F: ACCAGCCTCATCAACCAGAA	226

	R： GGTGCTGCAGGTCCTTAATG	
ACTA1	F： TCTTCCAGCCCTCCTTCATC<br>R： CTCTTTCTGCATGCGGTCAG	167
NPPA	F： GCATTCCAGCTCCTAGGTCA<br>R： CGCTTCTTCATTCGGCTCAC	165
Col1A1	F: GGCAAAGATGGACTCAACGG<br>R: ATCATCAGCCCGGTAGTAGC	210
COL3A1	F: GGCAAAGATGGAACCAGTGG<br>R: TCACCTCCAATCCCAGCAAT	188
ACTA2	F: GTTCCAGCCATCCTTCATCG<br>R: TGATCTTCATGGTGCTGGGT	200
PTEN	F: AGTCAGAGGCGCTATGTGTA<br>R: GTCGTGTGGGTCCTGAATTG	193
PDK4	F: TTGGCTGGTTTTGGTTACGG<br>R: CACCAGTCATCAGCCTCAGA	209
HMGB1	F: TGCTCAGAGAGGTGGAAGAC<br>R: CAGAAGAGGAAGAAGGCCGA	185
DKK1	F: GGTCTTTGTCGCGATGGTAG<br>R: CGGCTGGTAGTTGTCAATGG	214
ATF7	F: AAGAACTGTGAGGAGGTGGG<br>R: GACGTACAATGGTGGGTGTG	220
TLR-1	F： AACGTGAAGCTACAGGGTCA<br>R： TTTGGATGGGCAAAGCATGT	201
TLR-3	F： GAAGTACTTGACCTGGGCCT<br>R： AGGCTGGAATGGTGAAGGAG	213
TLR-4	F： ATCATTGGTGTGTCGGTCCT<br>R： CAGTCCTCATCCTGGCTTGA	164
TLR-6	F： AGTGAGTGGTGCCATTACGA<br>R： TTTGCTTTTCTCCTTGGGCC	183

TLR-9	F：CTCCGTGACAATTACCTGGC	197
	R：TTGGCCTTGGAAAAGAAGCC	
PRKCA	F: GCTGACTTTGGGATGTGCAA	165
	R: CCCGGCAAGCATTTCATACA	
PRKCB	F: CCGCCTGTACTTTGTGATGG	158
	R: GGTCACGGTAAATGATGCCC	
CYBB	F: ACCCTTCGCATCCATTCTCA	226
	R: ATTGGCCTGAGACTCATCCC	
GAPDH	F：CAAGAAGGTGGTGAAGCAGG	200
	R：CCACCCTGTTGCTGTAGCC	
miR-29a-3p	RT：GTCGTATCCAGTGCGTGTCGTGGAGTCG	70
	GCAATTGCACTGGATACGACTAACCGATTTC	
	F：TAGCACCATCTGAAATCGGTTA	
	R：GTGCGTGTCGTGGAGTC	
miR-103a-3p	RT：GTCGTATCCAGTGCGTGTCGTGGAGTCG	71
	GCAATTGCACTGGATACGACTCATAGCCCT	
	F：AGCAGCATTGTACAGGGCTATGA	
	R：GTGCGTGTCGTGGAGTC	
miR-182-5p	RT：GTCGTATCCAGTGCGTGTCGTGGAGTCG	72
	GCAATTGCACTGGATACGACAGTGTGAGTT	
	F：TTTGGCAATGGTAGAACTCACACT	
	R：GTGCGTGTCGTGGAGTC	
miR-135a-3p	RT：GTCGTATCCAGTGCGTGTCGTGGAGTCG	70
	GCAATTGCACTGGATACGACCGCCACGG	
	F：TATAGGGATTGGAGCCGTGGCG	
	R：GTGCGTGTCGTGGAGTC	
miR-424-5p	RT：GTCGTATCCAGTGCGTGTCGTGGAGTCG	70
	GCAATTGCACTGGATACGACTTCAAAACATG	

	F：CAGCAGCAATTCATGTTTTGAA	
	R：GTGCGTGTCGTGGAGTC	
U6	RT：GTCGTATCCAGTGCGTGTCGTGGAGTC GGCAATTGCACTGGATACGAC	160
	F：GTCCGCGTGCTCGCTTCGGCAGC	
	R：GTGCGTGTCGTGGAGTC	

RT: reverse transcription； F: forward； R: reverse
